# Dihydroartemisinin exerts antitumour activity by blocking SIRT2-IGFBP1-induced PI3K/AKT/mTOR signal transduction in liver cancer

**DOI:** 10.1038/s41598-026-49240-5

**Published:** 2026-04-20

**Authors:** Zhengbin Chai, Jingwen Liu, Jing Liu, Linfei Niu, Xiaoqing Wang, Chunyan Liu, Fabin Han

**Affiliations:** 1 Department of Clinical Laboratory Medicine, Public Health Clinical Center Affiliated to Shandong University, Shandong University, Shandong, 250013 People’s Republic of China; 2 Translational Research Laboratory for Stem Cell and Traditional Chinese Medicine, Innovation Institute for Traditional Chinese Medicine, Shandong University of Traditional Chinese Medicine, 4665 University Avenue, Jinan, 250355 Shandong People’s Republic of China; 3 College of Traditional Chinese Medicine, Shandong University of Traditional Chinese Medicine, Jinan, 250355 People’s Republic of China; 4 Jinan Clinical Research Center for Tissue Engineering Skin Regeneration and Wound Repair, The First Affiliated Hospital of Shandong First Medical University & Shandong Provincial Qianfoshan Hospital, Jinan, 250014 Shandong People’s Republic of China; 5 Key Laboratory of Biopharmaceuticals, Engineering Laboratory of Polysaccharide Drugs, National–Local Joint Engineering Laboratory of Polysaccharide Drugs, Shandong Academy of Pharmaceutical Science, Jinan, 250101 People’s Republic of China; 6 The Institute for Tissue Engineering and Regenerative Medicine/Key Laboratory of Health Commission of Shandong Province, Liaocheng University/Liaocheng People’s Hospital, Liaocheng, 252000 Shandong People’s Republic of China

**Keywords:** Liver cancer, SIRT2, IGFBP1, DHA, Cell proliferation, Cancer, Cell biology, Molecular biology, Oncology

## Abstract

The human NAD (+)-dependent deacetylase silent information regulator isoform 2 (SIRT2) is the cytoplasm-localized member of the sirtuin family, which has received increasing attention for its potential roles in cancer diagnosis and therapy. However, its role is still under debate, and its molecular mechanism remains unclarified. In this study, we thoroughly investigated the function and regulatory mechanism of SIRT2 in the tumorigenesis of liver cancer and found that SIRT2 is highly expressed in liver cancer tissues and is distributed mainly in the cytoplasm of liver cancer cells. The SIRT2 expression level is positively related to cell proliferation in vitro and tumour growth in vivo. We found that SIRT2 can upregulate IGFBP1 expression and subsequently activate the PI3K/AKT/mTOR signalling pathway and that IGFBP1 is essential for the tumour-promoting function of SIRT2 in liver cancer. Moreover, we characterized that dihydroartemisinin, one of the main active metabolites of artemisinin derivatives can inhibit liver cancer cell proliferation and liver tumour growth by promoting ubiquitin-dependent SIRT2 degradation and subsequently blocking SIRT2-IGFBP1-induced PI3K/AKT/mTOR signal pathway.

**Supplementary Information:**

The online version contains supplementary material available at 10.1038/s41598-026-49240-5.

## Introduction

Liver cancer is the third leading cause of cancer-related death worldwide, and more than 90% of cases with liver cancer are diagnosed as hepatocellular carcinoma ^1^. Surgical resection, local radiofrequency ablation, hepatic artery embolization, and liver transplantation are currently commonly used as treatment methods for patients with early liver cancer^2^. However, most patients are diagnosed at an advanced stage because of the lack of biomarkers at early stages, resulting in a high recurrence rate of liver cancer^3^. Despite the increasing choices of clinical therapies for liver cancer, only a few patients can benefit from these therapies, and the quality of life and survival rate of most patients have not been improved significantly. To investigate the molecular mechanism underlying liver cancer development can be helpful for identifying effective biomarkers for early diagnosis and drugs with low toxicity and high efficiency for cancer therapy.

The liver is a key organ for the maintenance of glucose and lipid homeostasis and the synthesis of secreted proteins and endocrine cytokines throughout the body. The insulin regulatory system plays an important role in physiological and pathological processes in the human body. Dysfunction of insulin-like growth factors (IGFs) is closely related to the occurrence and development of liver cancer, as IGFs are among the growth factors most strongly related to the tumorigenesis of liver cancer^4^.

Silent information regulator isoform 2 (SIRT2) is the only member of the SIRT family that is distributed mainly in the cytoplasm^5^, and it plays an important role in regulating multiple physiological and pathological signal transduction pathways, especially those involved in the pathogenesis of various types of cancer^6^. SIRT2 is reportedly involved in several cellular signalling pathways involved in cancer, including the nuclear factor kappa B (NF-κB)/signal transducers and activators of transcription 3 (STAT3), epidermal growth factor receptor (EGFR), and PI3K/mTOR pathways. A recent study revealed that rapamycin treatment increased deacetylase activity of SIRT2 towards FKBP12. The FKBP12 protein deacetylated by SIRT2 is then associated with mTOR to inactivate the mTOR pathway to inhibit cell cycle progression and cancer growth^7,8^. However, the downstream regulatory mechanism of SIRT2 in the development of liver cancer, which may be helpful for clinical therapy of liver cancer, is still obscure and needs further study.

In this study, we focused on the IGF system to identify a target protein of SIRT2 in the development of liver cancer and found that SIRT2 can exhibit tumour-promoting activity by upregulating insulin-like growth factor binding protein 1 (IGFBP1), a secreted protein that can specifically bind to IGFs and regulate cellular mitogenesis and metabolism. Early research revealed that, compared with healthy individuals, patients with liver cancer have a higher of IGFBP1^9^, which can regulate cell metastasis, angiogenesis, cell proliferation, and energy metabolism^10–12^. However, some studies indicated that the role of IGFBP1 remains controversial. In this study, we found that IGFBP1 expression can be positively regulated by SIRT2 and that knockdown of IGFBP1 can impair the ability of SIRT2 to promote cell proliferation and signal transduction, indicating that IGFBP1 plays a key role downstream of SIRT2 in the development of liver cancer.

SIRT2 is considered a promising drug target because many selective inhibitors of SIRT2 have been reported to exhibit excellent antitumour activity^13–17^. The exploration of SIRT2-targeting drugs with low toxicity and high efficiency is a promising strategy for improving cancer therapy. Dihydroartemisinin (DHA), the main active metabolite of artemisinin derivatives, has been reported to exhibit strong antibacterial, antiviral, and antitumour effects in a variety of human cancers^18–20^. Recent studies have shown that DHA exhibits strong antitumour activity in several kinds of cancers, including liver cancer, lung cancer, and breast cancer ^21,22^. Most recently, a study reported that DHA inhibited the proliferation and migration of HepG2 and HuH-7 liver cancer cells by reducing ATP synthase production via the CaMKK2/NCLX signalling pathway^23^. In the present study, we found that DHA can suppress liver cancer cell proliferation by inhibiting SIRT2 expression via its ubiquitin-dependent degradation and blocking SIRT2-IGFBP1-induced signal transduction.

## Materials and methods

### Reagents

All the antibodies used in this study were obtained from commercial companies. The detailed information, including the manufacturer and catalogue number, is listed as follows: anti-GAPDH rabbit monoclonal antibody (mAb) (#5174; CST), anti-Flag (D6W5B) rabbit mAb (14793S; CST), anti-IGFBP1 (D4E9T) rabbit mAb (#31,025; CST), anti-mTOR (7C10) rabbit mAb (#2983; CST), anti-phospho-mTOR (Ser2448) (D9C2) rabbit mAb (#5536; CST), anti-AKT (C67E7) rabbit mAb (#4691; CST), anti-phospho-Akt (Ser473) (D9E) rabbit mAb (#4060; CST), anti-PI3 Kinase p85 rabbit mAb (#4292; CST), anti-phospho-PI3 Kinase p85 (Tyr458)/p55 (Tyr199) (E3U1H) rabbit mAb (#17,366; CST), anti-SIRT2 (D4O5O) rabbit mAb (#12,650; CST), anti-acetyl-α-tubulin (Lys40) rabbit antibody (#3971; CST), anti-α-tubulin rabbit mAb (#2125; CST), anti-acetyl-NF-κB p65 (Lys310) antibody (#3045; CST), anti- NF-κB p65 rabbit mAb (#8242; CST), anti-caspase-3 rabbit mAb (#9662; CST) , anti-cleaved caspase-3 rabbit mAb (#9661; CST) , anti-PCNA rabbit mAb (#13,110; CST), and horseradish peroxidase (HRP)-conjugated anti‐rabbit IgG (#7074; CST).

### Liver cancer cell lines

HepG2(CTCC-00-0014) and Huh-7(CTCC-003-0019) cell lines used in this study were purchased from Meisen cell technology Co. Ltd., Meisen Chinese Type Culture Collection (MeisenCTCC, Hangzhou, China) and are authenticated with STR profiling.

### Collection of tissue specimens

The liver cancer tissues and corresponding adjacent tissues used in this study were collected and pathologically confirmed at the First Affiliated Hospital of Shandong First Medical University, and tissue arrays (HLivH160CS02) were purchased from Shanghai OUTDO Biotech Co., Ltd. (Shanghai, China).

### In vivo xenograft tumour experiments

In this study, 5-week-old specific pathogen-free (SPF) BALB/c nude mice (half male and half female) were purchased from Beijing Vital River Laboratory Animal Technology Company and raised at the Association for Assessment and Accreditation of Laboratory Animal Care (AAALAC)-accredited SPF facility. For construction of the Huh7 cell line with SIRT2 overexpression, the full open reading frame of SIRT2 (NM_012237) was inserted into the doxycycline (DOX)-inducible Tet-on promoter GV308 expression vector, which contained a doxycycline-inducible promoter, a puromycin resistance cassette for screening, and a flag tag, and the resulting construct was packaged into lentiviral particles (GeneChem, Shanghai, China). The reconstructed SIRT2-expressing vector was subsequently transfected into Huh7 cells, which were subsequently screened with 5 μg/mL puromycin for 24 h to establish Huh7 cells stably expressing SIRT2 (the cells stably expressing SIRT2 need to be screened with 5 μg/mL puromycin for 24 h before use), which was then treated with 5 μg/mL doxycycline for 48 h before cells are harvested. Finally, 5 × 10^6^ SIRT2-expressing Huh7 cells were collected, suspended in 200 μL of phosphate-buffered saline (PBS) and then injected into the right dorsal flanks of BALB/c nude mice. The injected mice were subsequently fed a diet containing 500 mg/kg doxycycline buffer for 5 weeks. At the end of the experiment, mice were anesthetized by inhalation of isoflurane (2–5%) and subsequently euthanized by cervical dislocation, and the formed tumours were collected for subsequent analysis. Empty GV308 vector-transfected Huh7 cells were used as the control group. Tumor measurements were performed in a blinded fashion.

### Extraction of total protein and western blot analysis

One hundred micrograms of solid liver cancer tissues or corresponding adjacent tissues were homogenized in 500 μL of cell lysis buffer (KeyGEN BioTECH, China) containing 1% phenylmethylsulfonyl fluoride (Beyotime, China) and 1% protease and phosphatase inhibitor cocktail (New Cell & Molecular Biotech Co., Ltd., Suzhou, China), and the lysate was centrifuged at 12,000 ×*g* for 15 min at 4 °C. The protein concentration in the supernatant was subsequently measured with a Bradford Protein Assay Kit (Beyotime, China). Finally, sodium dodecyl sulfate–polyacrylamide gel electrophoresis (SDS‒PAGE) protein loading buffer was added to the lysate, and the mixture was vortexed. After denaturation at 100 °C for 10 min, the total protein mixture was aliquoted and stored in an ultra-low temperature freezer for further study.

The protein samples (30 μg/well) were separated by SDS‒PAGE and then transferred onto a 0.22-μm pore polyvinylidene fluoride (PVDF) membrane (Millipore, USA) at 300 mA. TBS containing 5% skim milk was used as the blocking buffer for incubation of the PVDF membrane containing total protein, which was subsequently washed with TBS three times to remove excess blocking buffer and then incubated with a primary antibody (diluted 1:1000 in Primary Antibody Dilution Buffer) overnight at 4 °C. Finally, the PVDF membrane was incubated with an HRP-conjugated secondary antibody (diluted 1:10,000 in blocking buffer) at room temperature for 1 h, and images were acquired with a Bio-Rad ChemiDoc MP Imaging System (Bio-Rad, USA).

### Immunocytochemical analysis

In this study, Huh7 cells were seeded on glass slides in a 24-well plate and treated according to the requirements of the experiment. Then, the cells were washed with PBS to remove the complete DMEM, fixed with 4% neutral paraformaldehyde (Servicebio, Wuhan, China) and permeabilized with 0.2% Triton X-100 (Solarbio, Beijing, China). After being washed with PBS 3 times, the cells were incubated with a primary antibody (diluted according to the titre of the primary antibody), an immunofluorescent secondary antibody (Alexa Fluor 488-conjugated goat anti-rabbit IgG), and 4′,6-diamidino-2-phenylindole (DAPI, Sigma‒Aldrich, USA) to stain nuclei. Finally, the immunofluorescence signals were observed with a Zeiss fluorescence microscope.

### Immunohistochemical analysis

Immunohistochemistry was performed to determine the distribution of SIRT2 in liver cancer tissues. A tissue microarray containing 14 liver cancer tissue samples and 14 corresponding adjacent nontumor liver tissue samples was obtained commercially from Shanghai OUTDO Biotech Co., Ltd. (HLivH160CS02, Shanghai, China). Detailed clinicopathological information of the corresponding patients, including age, clinicopathological stage at diagnosis, lesion site, and degrees of distal metastasis and infiltration, was provided by the manufacturer and is available. The protocol for immunohistochemistry was as follows: in brief, the tissue microarray sections were deparaffinized with dimethylbenzene, rehydrated through an alcohol gradient and heated for antigen retrieval. After being washed with PBS, the tissue microarray sections were treated with H_2_O_2_ to quench endogenous peroxidase and oxidoreductase activity and incubated first with an anti-SIRT2 primary antibody (diluted 1:300 in PBS) overnight at 4 °C and then with Alexa Fluor 488-conjugated goat anti-rabbit IgG, following standard procedures, at room temperature for 1 h. Finally, the nuclei were stained with DAPI (Sigma‒Aldrich, USA) at room temperature for 10 min. The tissue microarray slide was mounted with ProLong Gold Antifade Mountant, and the immunoreactive signals were observed with a fluorescence microscope (Carl Zeiss, Jena, Germany).

### Cell proliferation and colony formation assays

In this study, cell proliferation and viability were measured via a Cell Counting Kit-8 (CCK8, Dojindo, Japan). The procedure was as follows: 8000 cells/well were seeded into 96-well plates and cultured for 24 h. Then, the cells were transfected with plasmid/siRNA or treated with drugs according to the manufacturer’s instructions. Finally, the culture medium was discarded, and a mixture of CCK8 buffer and culture medium (100 μL of complete culture medium mixed with 10 μL of CCK8 buffer) was added to the wells, and incubation was continued for another 2 h. The absorbance was then measured at 450 nm via a SpectraMax 190 microplate reader (Molecular Devices, USA). The relative cell viability was calculated by normalizing the absorbance of the experimental group to that of the control group.

A colony formation assay was also performed to verify the results of the CCK8 assay. For this assay, 2 × 10^3^ liver cancer cells (Huh7 or HepG2) were collected, suspended in 6 mL of complete DMEM, and then seeded into a round cell culture dish with a diameter of 6 cm and incubated for approximately 10‒15 days (according to the cell line). Finally, the cells were washed with PBS to remove the culture medium, fixed with methanol for 10 min and stained with 0.25% crystal violet for 10 min. The number of cell colonies was statistically analysed after washing with PBS.

### RNA extraction and quantitative real-time PCR (RT-qPCR)

Total RNA was extracted from cultured cells using TRIzol reagent (Vazyme, China) according to the manufacturer’s instructions. The concentration and quality of the extracted RNA were measured using a NanoDrop spectrophotometer. Subsequently, 1 μg of total RNA was reverse-transcribed into cDNA using the HiScript II Q RT SuperMix for qPCR (Vazyme, China). Quantitative real-time PCR was performed using the ChamQ SYBR qPCR Master Mix (Vazyme, China) on a real-time PCR detection system. GAPDH was used as the internal reference gene. The relative mRNA expression levels were calculated using the 2^-ΔΔCt^ method. All experiments were performed in triplicate.

### Statistical analyses

Differences between groups were analyzed via two-tailed Student’s *t*-test (means ± SD). Before applying the t-test, the Shapiro–Wilk test was used to verify the normal distribution of the data, and Levene’s test was used to verify the homogeneity of variance. Differences were considered significant if *p* < 0.05 and highly significant if *p* < 0.01.

## Results

### SIRT2 is highly expressed in liver cancer tissues and is localized mainly in the cytoplasm

**Fig. 1:**
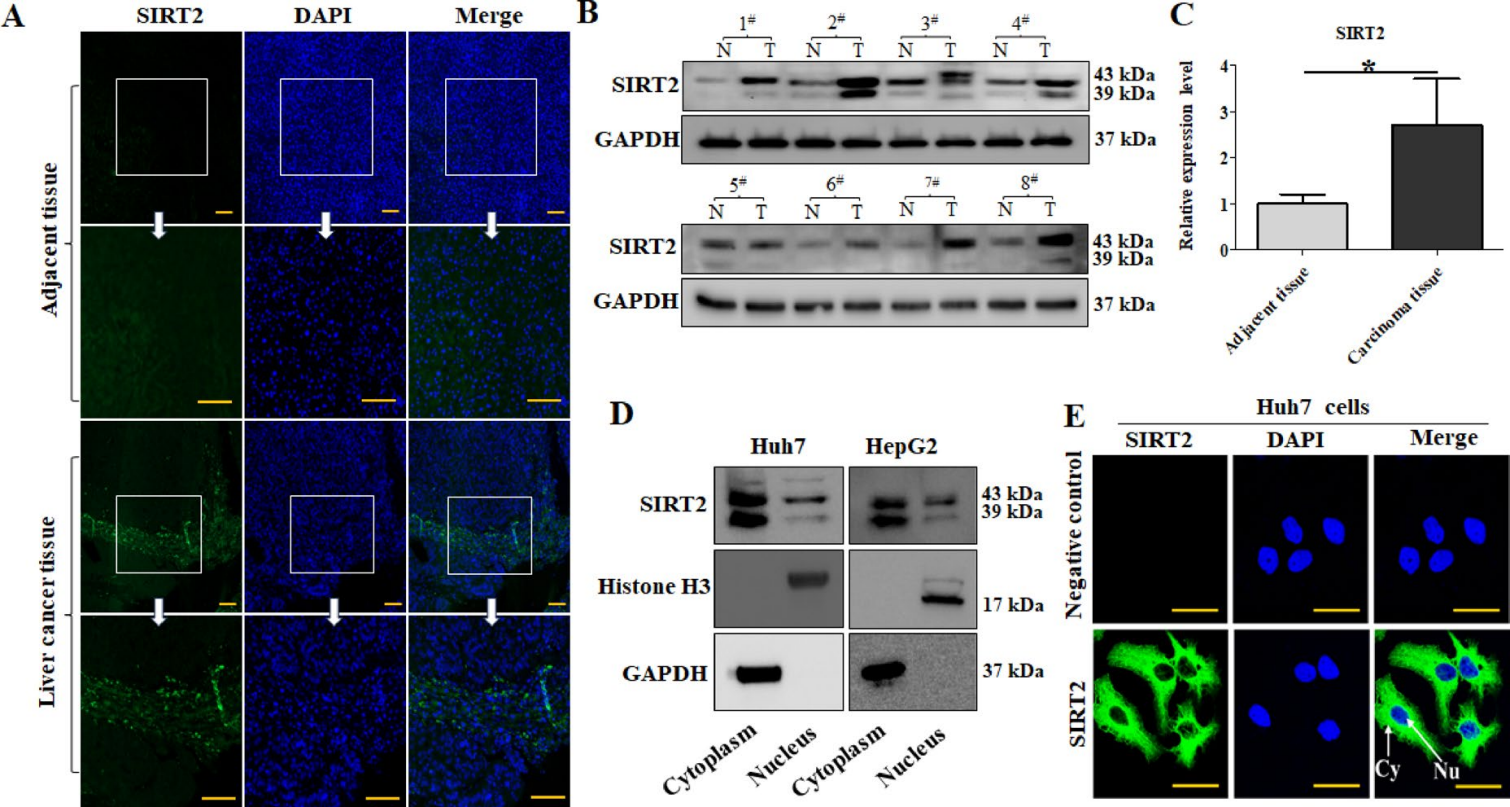
Tissue expression pattern and subcellular localization of SIRT2 in liver cancer cells. (**A**), Immunofluorescence staining was used to determine the distribution of SIRT2 in liver cancer tissues and the corresponding adjacent nontumor tissues. These tissue sections were purchased from Shanghai OUTDO Biotech Co, scale bar = 100 μm. (**B**), Western blotting was performed to confirm the expression level of SIRT2 in both the liver cancer tissues and the corresponding adjacent nontumor tissues. These paired tissues were obtained from 8 different liver cancer patients, and GAPDH was selected as the internal reference to normalize the relative expression level of SIRT2. (**C**), Statistical analysis of the relative expression level of SIRT2 in **B**. (**D**), Cytoplasmic and nuclear proteins were extracted from both HepG2 and Huh7 cells and analysed via western blotting to determine the subcellular distribution of SIRT2. The two close bands detected by the SIRT2 antibody represent the two main isoforms of human SIRT2. (**E**), Immunocytochemistry was performed to visually confirm the cytoplasmic localization of SIRT2 using Huh7 cells as a representative model, scale bar = 20 μm. Data are presented as the mean ± SD (n = 3 independent experiments), *p < 0.05 for three independent experiments; ** *p* < 0.01 for three independent experiments; Student’s t test.

To study the role of SIRT2 in liver cancer, its tissue distribution and expression profile were measured in liver cancer tissues and adjacent tissues, for it is difficult to obtain completely healthy human liver samples, pathologically confirmed adjacent non-tumor tissues were used as the normal baseline control to evaluate SIRT2 expression. Immunohistochemistry was used to detect the tissue distribution of SIRT2, and the results showed that SIRT2 was distributed mainly in carcinoma tissues and was only weakly distributed in the corresponding adjacent tissues (Fig. 1A). The SIRT2 expression pattern was also verified via western blotting, which revealed that SIRT2 was more highly expressed in liver cancer tissues than that in the corresponding adjacent tissues (Fig. 1B, C), consistent with the immunohistochemical results. Collectively, these results show that SIRT2 is highly expressed in liver cancer tissues, suggesting that it may contribute to the development of liver cancer.

To further explore the function of SIRT2, nuclear and cytoplasmic protein fractions were extracted from both HepG2 and Huh7 cells. Western blot analysis revealed that SIRT2 was localized mainly in the cytoplasm in both cell lines, with only a small amount detected in the nucleus (Fig. 1D). This subcellular localization was further verified by immunocytochemistry using Huh7 as a representative cell line, which visually demonstrated that SIRT2 was distributed mainly in the cytoplasm (Fig. 1E). These results indicate that SIRT2 is distributed mainly in the cytoplasm of liver cancer cells to play its role in the initiation of tumorigenesis. HepG2 (wild-type p53) and Huh7 (mutant p53) cell lines were used for subsequent assays because they represent different genetic backgrounds of liver cancer. Both cell lines show stable baseline SIRT2 expression, which makes them suitable in vitro models for evaluating protein degradation.

### SIRT2 can positively regulate liver cancer cell proliferation and tumour development

**Fig. 2:**
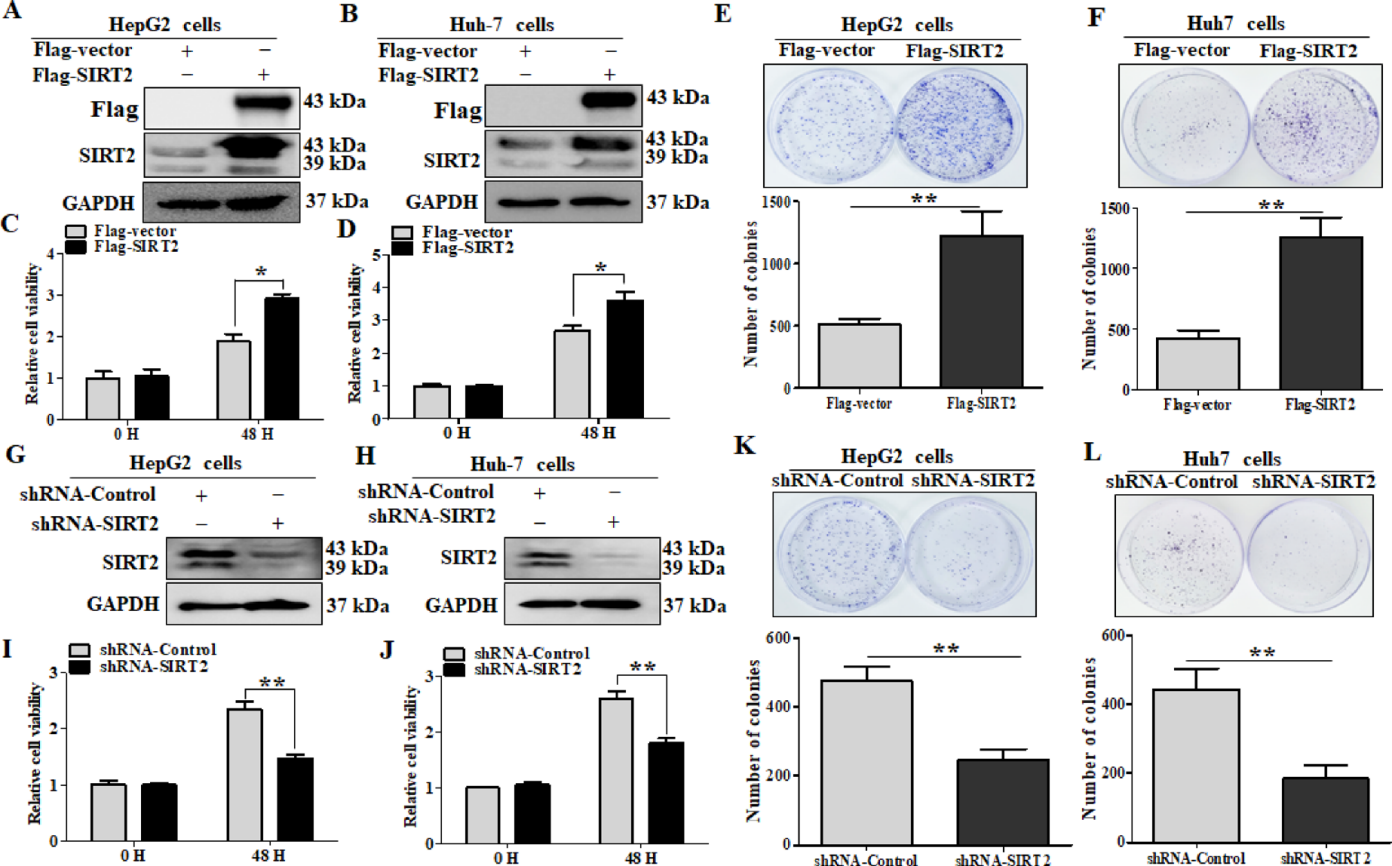
Effects of SIRT2 on the proliferation and colony formation of liver cancer cells. (**A**, **B**) The transduction efficiency was confirmed via western blotting after SIRT2-overexpressing lentiviral vectors were transduced, and protein expression was induced with doxycycline for 48 h. (**C**, **D**), The proliferation of HepG2 and Huh7 cells was evaluated via a CCK8 assay after SIRT2 was overexpressed. (**E**, **F**), The proliferation ability of HepG2 and Huh7 cells was evaluated via a colony formation assay after SIRT2 was overexpressed. (**G**, **H**), The expression level of SIRT2 was measured via western blotting after SIRT2 was knocked down via lentiviral transduction of a shRNA vector to verify the transduction efficiency. (**I**, **J**), The proliferation of HepG2 and Huh7 cells after SIRT2 expression was knocked down was evaluated via a CCK8 assay. (**K**, **L**), The proliferation of HepG2 and Huh7 cells after SIRT2 expression was knocked down was evaluated via a colony formation assay. The two close bands detected by the antibody represent the two main isoforms of SIRT2. Data are presented as the mean ± SD (n = 3 independent experiments),    * *p* < 0.05 for three independent experiments; ** *p* < 0.01 for three independent experiments; Student’s t test.

Although our results indicate that SIRT2 is localized mainly in the cytoplasm of liver cancer cells, its function and regulatory mechanism still need further exploration. To determine the role of SIRT2 in liver cancer cell proliferation, CCK8 and colony formation assays were performed. The results revealed that when SIRT2 was overexpressed (Fig. 2A, B), the proliferation of both HepG2 cells and Huh7 cells was promoted, as determined via a CCK8 assay (Fig. 2C, D). These results were also confirmed via a colony formation assay, which revealed that the overexpression of SIRT2 promoted colony formation in both HepG2 cells and Huh7 cells (Fig. 2E, F).

To further validate the function of SIRT2, it was also knocked down by shRNA transduction in both HepG2 cells and Huh7 cells (Fig. 2G, H). The results showed that suppressing SIRT2 expression inhibited cell proliferation (Fig. 2I, J) and colony formation (Fig. 2K, L) in both tested cell lines, consistent with the above results. These results indicate that the expression level of SIRT2 is positively related to the proliferative activity of HepG2 and Huh7 cell lines.

**Fig. 3:**
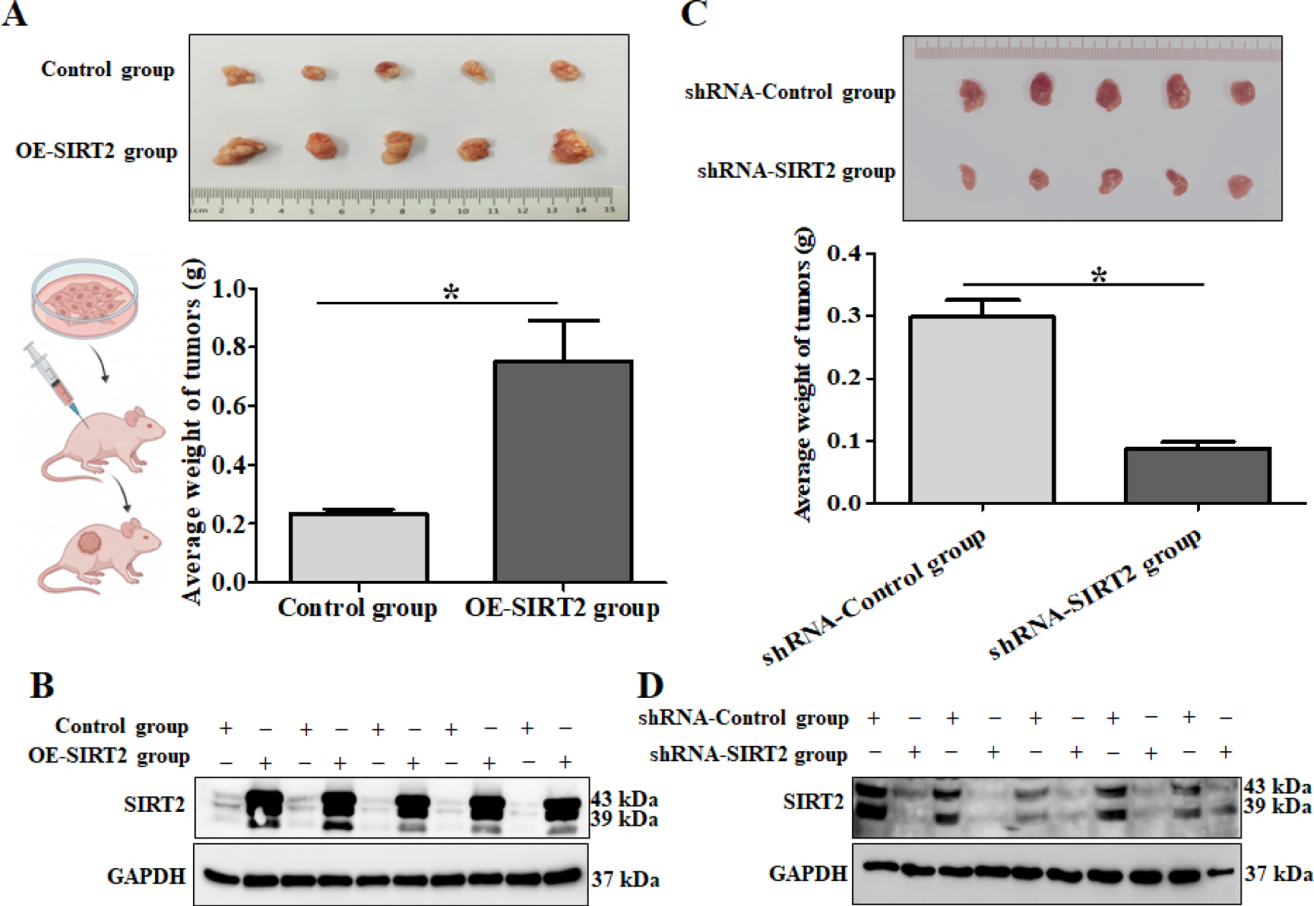
Xenograft experiments were performed to study the role of SIRT2 in vivo in BALB/c nude mice. (**A**), A total of 5 × 10^6^ Huh7 cells stably overexpressing SIRT2 were injected subcutaneously into the dorsal flanks of the mice in the experimental group, and an equal number of empty vector-transfected Huh7 cells were injected subcutaneously into the dorsal flanks of the mice in the control group. Tumours were harvested 6 weeks after cell injection, and tumour weights were measured and statistically analysed. (**B**), Total protein was extracted from the xenograft tumours, and SIRT2 overexpression was measured via western blotting with an anti-Flag antibody. (**C**), A total of 5 × 10^6^ SIRT2 shRNA-SIRT2-transduced Huh7 cells were injected subcutaneously into the dorsal flanks of the mice in the experimental group, and an equal number of empty vector-transfected Huh7 cells were injected into the mice in the control group. Tumours were harvested 7 weeks after cell injection, and the weights were measured and statistically analysed. (**D**), Total protein was extracted from the xenograft tumours, and the expression level of SIRT2 was measured via western blotting. The two close bands detected by the antibody represent the two main isoforms of SIRT2. Data are presented as the mean ± SD (n = 3 independent experiments), * *p* < 0.05 for three independent experiments; ** *p* < 0.01 for three independent experiments; Student’s t test.

The role of SIRT2 in liver cancer was also studied in vivo via a xenograft experiment. In this study, the Huh7 cell line was selected for the in vivo xenograft model because of its high tumorigenicity and aggressive growth phenotype in nude mice compared to HepG2. Both Huh7 cells with stable SIRT2 overexpression and Huh7 cells with stable SIRT2 knockdown were injected into BALB/c nude mice to determine the role of SIRT2 in tumorigenesis. It was found that the overexpression of SIRT2 promoted tumour growth (Fig. 3A, B) and that the knockdown of SIRT2 inhibited tumour growth (Fig. 3C, D), the knockdown efficiency of SIRT2 was also confirmed at the mRNA level by RT-qPCR (Supplementary Figure S3A), which is consistent with the results of the in vitro studies. These results indicate that SIRT2 can positively regulate liver cancer cell proliferation and tumour growth both in vitro and in vivo. Further study of the molecular mechanism of SIRT2 in liver cancer may aid in cancer diagnosis, anticancer therapy, and anticancer drug design.

### IGFBP1 can be upregulated by SIRT2 and promote cell proliferation by regulating PI3K/AKT/mTOR phosphorylation

**Fig. 4:**
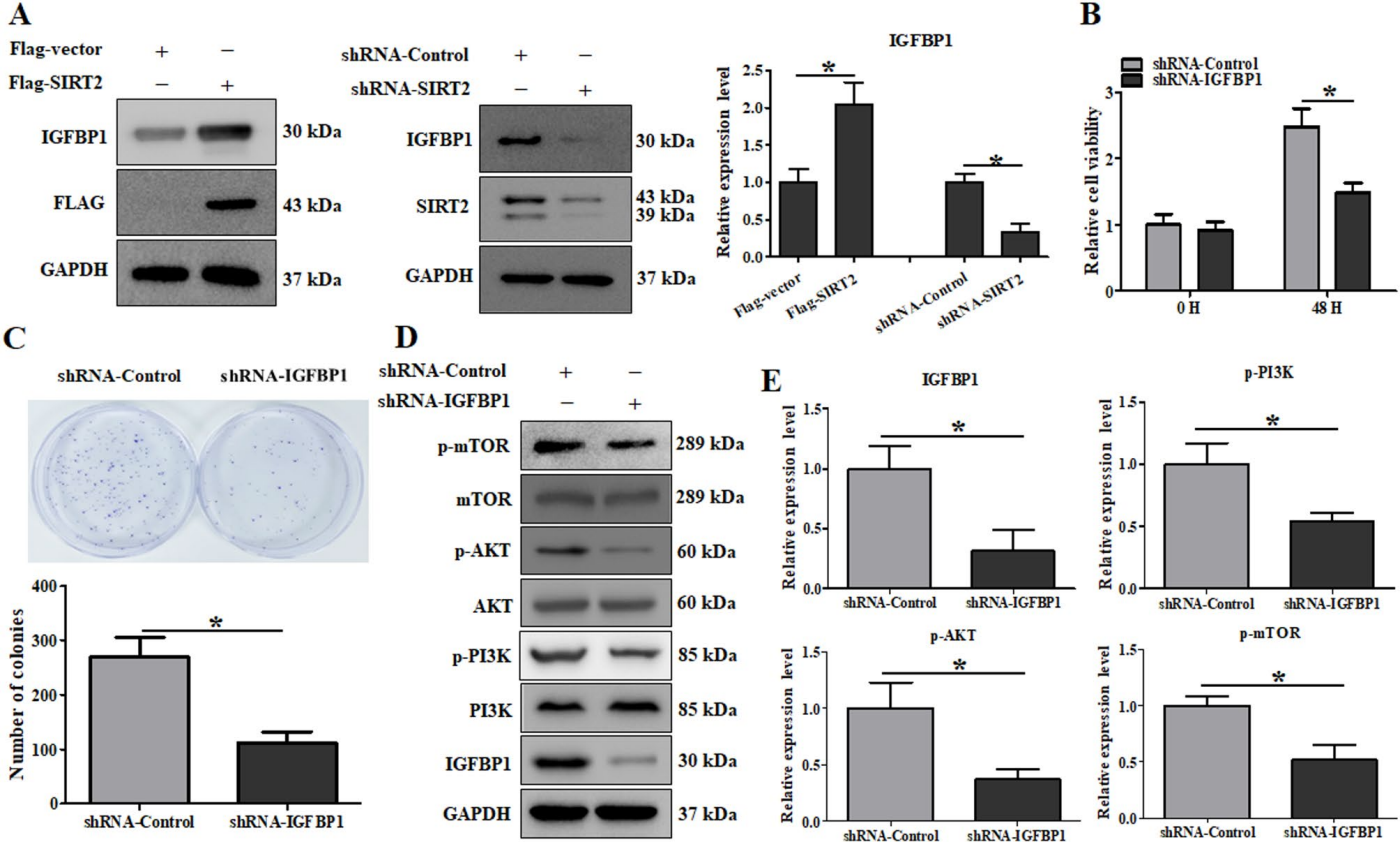
Function and regulatory mechanism of IGFBP1 in liver cancer cells. (**A**), The regulation of IGFBP1 expression by SIRT2 in SIRT2-overexpressing and SIRT2-knockdown liver cancer cells. (**B**), The proliferation of HepG2 cells was evaluated via a CCK8 assay after IGFBP1 expression was knocked down. (**C**), Cell proliferation was verified via a colony formation assay in HepG2 cells after IGFBP1 expression was knocked down. (**D**), Western blotting was used to evaluate the effects of IGFBP1 on the levels of p-PI3K, p-AKT and p-mTOR. (**E**), Statistical analysis of the relative expression levels of IGFBP1, p-PI3K, p-AKT and p-mTOR in D. Data are presented as the mean ± SD (n = 3 independent experiments), * *p* < 0.05 for three independent experiments; ** *p* < 0.01 for three independent experiments; Student’s t test.

To study the molecular mechanism of SIRT2 in the tumorigenesis of liver cancer in detail, proteomic analysis was performed, and 12 differentially expressed proteins were identified: 4 downregulated proteins NDE-like 1 (NDEL1), suppressor of morphogenesis in genitalia 1 (SMG1), alpha/beta-hydrolase domain-containing 6 (ABHD6), and Laminin subunit beta-2 (LAMB2) and 8 upregulated proteins solute carrier family 2 member 1 (SLC2A1), IGFBP1, proliferating cell nuclear antigen (PCNA), Visinin-like 1 (VSNL1), insulin-like growth factor 2 mRNA binding protein 3 (IMP3), Zinc Finger MYND-Type Containing 8 (ZMYND8), Acyl CoA synthase long-chain family member 1 (ACSL1), and mitochondrial cytochrome c oxidase subunit-1 (MT-CO1) (Figure S1). Western blotting revealed that SIRT2 can upregulate the expression of IGFBP1, which is positively related to the SIRT2 expression level (Fig. 4A). Furthermore, RT-qPCR analysis demonstrated that knockdown of SIRT2 significantly decreased the mRNA level of IGFBP1, whereas SIRT2 overexpression increased the mRNA level of IGFBP1 (Supplementary Figure S2). This indicates that SIRT2 regulates IGFBP1 expression at the transcriptional level.

The function of IGFBP1 was also studied in liver cancer cells via CCK8 and colony formation assays. The results showed that when IGFBP1 was knocked down by shRNA transduction in liver cancer cells, the cell proliferation (Fig. 4B) and colony formation abilities (Fig. 4C) were significantly reduced, the knockdown efficiency of IGFBP1 was also confirmed at the mRNA level by RT-qPCR (Supplementary Figure S3B). These results indicate that, similar to SIRT2, IGFBP1 can promote cell proliferation in vitro and may be a key downstream protein through which SIRT2 functions in the initiation of liver cancer development.

To further study the molecular mechanism of IGFBP1 in the tumorigenesis of liver cancer, a set of related proteins was analysed via western blotting, and the results revealed that the knockdown of IGFBP1 decreased the levels of p-PI3K, p-AKT, and p-mTOR (Fig. 4D, E), indicating that IGFBP1 may play its role in the development of liver cancer via the PI3K-AKT-mTOR signalling pathway.

### Knockdown of IGFBP1 can impair SIRT2-induced liver cancer cell proliferation and PI3K/AKT/mTOR phosphorylation

**Fig. 5:**
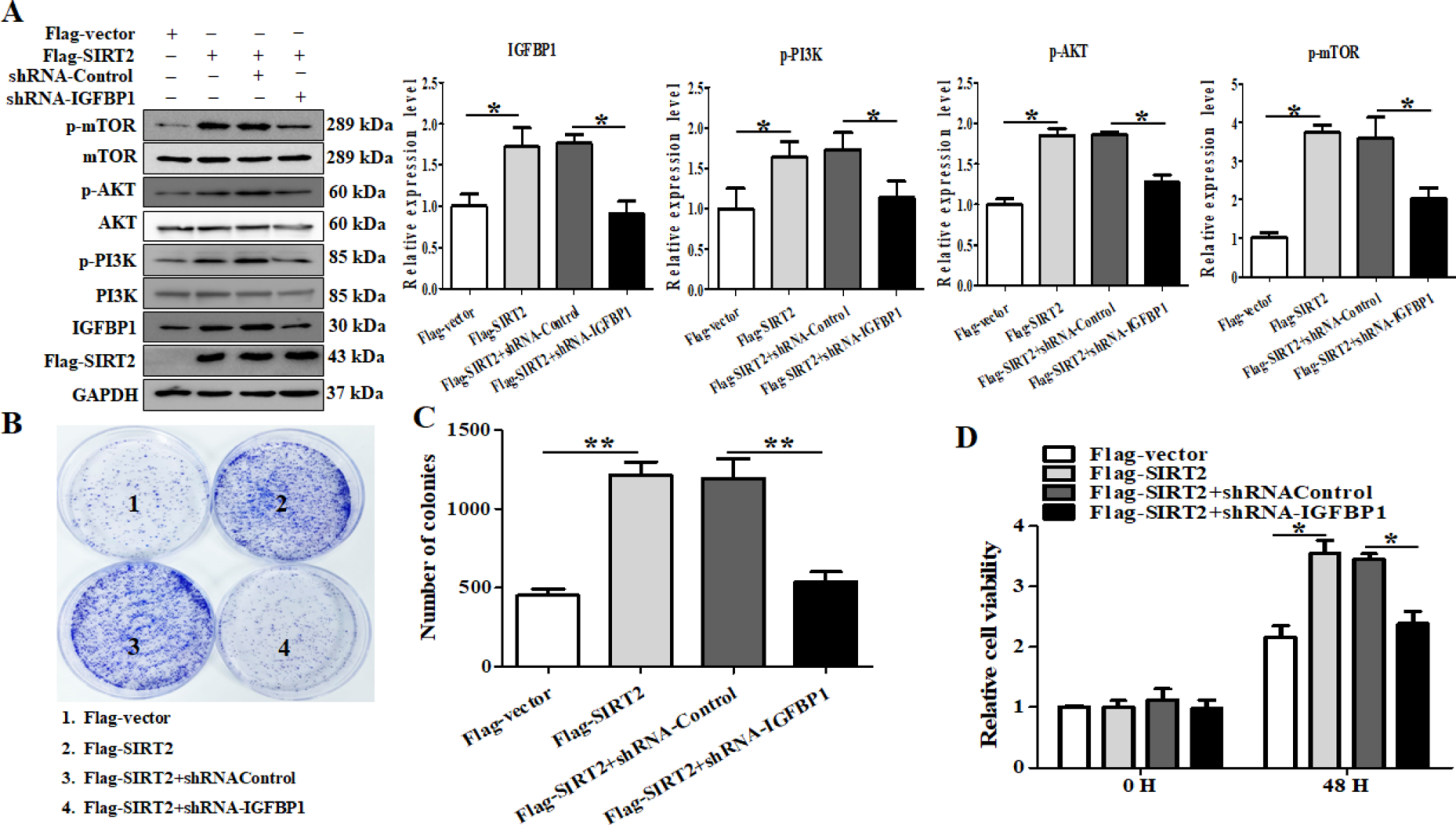
Function and molecular mechanism of SIRT2 and IGFBP1 in the tumorigenesis of liver cancer in HepG2 cells. (**A**), A rescue experiment was performed in HepG2 cells with doxycycline-induced SIRT2 overexpression with or without IGFBP1-specific shRNA transduction, and the levels of p-PI3K, PI3K, p-AKT, AKT, p-mTOR, and mTOR were measured and statistically analysed via western blotting. Empty vector-transfected HepG2 cells were used as the control group, an anti-Flag antibody was used to detect the overexpression of SIRT2. (**B**), A colony formation assay was performed to confirm the role of IGFBP1 in SIRT2-overexpressing HepG2 cells. **C**, Statistical analysis of the colony formation assay results in (**B**. **D**), A CCK8 assay was used to confirm the role of IGFBP1 in SIRT2-overexpressing HepG2 cells. Data are presented as the mean ± SD (n = 3 independent experiments), * *p* < 0.05 for three independent experiments; ** *p* < 0.01 for three independent experiments; Student’s t test.

To explore the roles of and the relationship between SIRT2 and IGFBP1 in the tumorigenesis of liver cancer, a rescue experiment was performed in HepG2 cells, in which IGFBP1-specific shRNA was transfected into HepG2 cells with doxycycline-induced SIRT2 overexpression. The results revealed that knockdown of IGFBP1 partially reversed SIRT2 overexpression-induced PI3K, AKT and mTOR phosphorylation (Fig. 5A). Colony formation and CCK8 assays were performed to confirm the role of IGFBP1 in SIRT2-overexpressing HepG2 cells, and the results of the colony formation assay revealed that knockdown of IGFBP1 abolished the colony formation ability of SIRT2-overexpressing liver cancer cells (Fig. 5B, C). A CCK8 assay was also used to verify the results and revealed that knockdown of IGFBP1 suppressed SIRT2 overexpression-induced liver cancer cell proliferation (Fig. 5D). These results suggest that IGFBP1 is essential for the ability of SIRT2 to promote cell proliferation and regulate PI3K/AKT/mTOR signal transduction during the development of liver cancer.

### DHA exerts an antitumour effect by suppressing the activity of the SIRT2/PI3K/AKT signalling pathway through the promotion of SIRT2 ubiquitination-mediated degradation in liver cancer cells

**Fig. 6:**
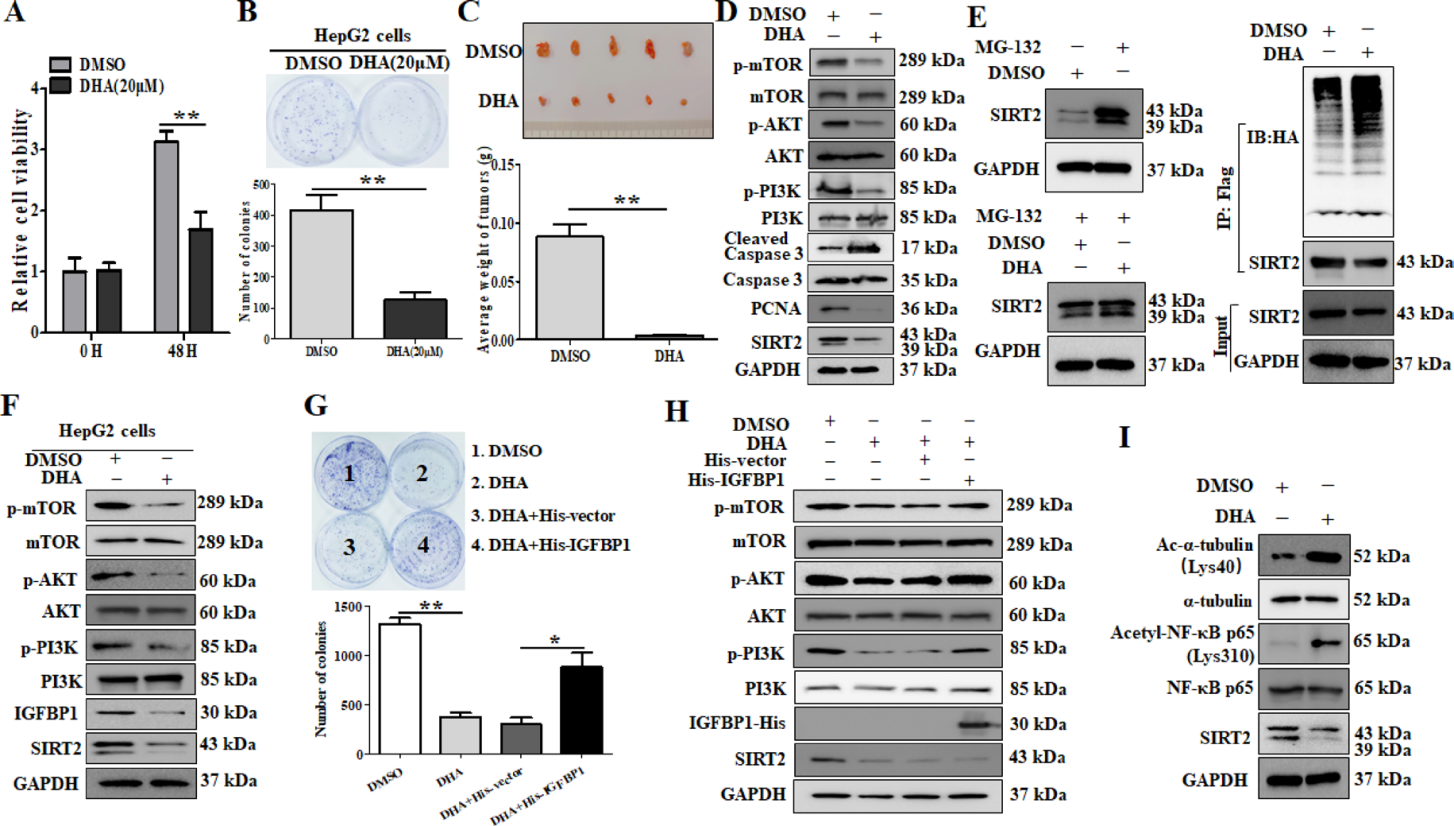
Role and regulatory mechanism of DHA in the tumorigenesis of liver cancer. A, A CCK8 assay was used to evaluate the effect of DHA on the proliferation of HepG2 cells. B, A colony formation assay was used to confirm the effect of DHA on the proliferation of HepG2 cells. C, SIRT2-overexpressing Huh7 cells (5 × 10^6^) were injected subcutaneously into the dorsal flanks of nude mice to establish a tumour-bearing model. After 3 weeks, the tumour-bearing nude mice were orally gavaged with DHA (80 mg/kg/d) for 14 days, after which the tumour weights were measured and statistically analysed. D, Western blotting was used to measure the protein levels of p-mTOR, mTOR, p-AKT, AKT, p-PI3K, PI3K, cleaved caspase-3, caspase-3, PCNA, and SIRT2 in the xenograft tumor tissues from the control (DMSO) and DHA-treated mice. E, HepG2 cells were treated with DMSO, MG132 (10 μM) alone, or pre-treated with MG132 followed by DHA treatment. SIRT2 protein levels were measured by western blotting to confirm the proteasome-dependent degradation (bottom). HA-ubiquitin-overexpressing plasmids were transfected into HepG2 cells stably overexpressing SIRT2, which were then treated with DHA (20 μM) for 24 h. CoIP was subsequently performed to evaluate the ubiquitin-dependent degradation of SIRT2 induced by DHA (top). F, Western blotting was used to study the effects of DHA on the protein levels of SIRT2, IGFBP1, p-PI3K, PI3K, p-AKT, AKT, p-mTOR, and mTOR. G, A rescue experiment was performed to study the function of IGFBP1 in the DHA-induced suppression of cell proliferation via a colony formation assay. H, Western blotting was used to study the function of IGFBP1 in DHA-induced signal transduction through the measurement of protein levels, including p-PI3K, PI3K, p-AKT, AKT, p-mTOR, and mTOR. I, Western blotting was used to measure the protein levels of acetyl-α-tubulin (Lys40), total α-tubulin, acetyl-NF-κB p65 (Lys310), and total p65 in liver cancer cells treated with DHA. Data are presented as the mean ± SD (n = 3 independent experiments), * Indicates p < 0.05 for three independent experiments; ** *p* < 0.01 for three independent experiments; Student’s t test.

DHA is a drug with low toxicity and high efficiency that has been reported to exhibit antitumour activity in various types of cancer, including liver cancer; however, its molecular mechanism is still obscure. In this study, the function of DHA in liver cancer was fully studied both in vitro and in vivo. The results showed that DHA inhibited cell proliferation and colony formation in vitro (Fig. 6A, B) and tumour growth in vivo (Fig. 6C), consistent with earlier reports. To further evaluate the molecular alterations in vivo, western blotting was performed using the xenograft tumor tissues. The results showed that DHA treatment decreased the expression of SIRT2 and the proliferation marker PCNA, increased the apoptosis marker cleaved caspase-3, and inhibited the phosphorylation of PI3K, AKT, and mTOR in the tumors without altering their total protein expressions (Fig. 6D). The molecular mechanism of DHA was further investigated. To determine whether the degradation of SIRT2 is ubiquitin-proteasome dependent, HepG2 cells were treated with the proteasome inhibitor MG132. The western blot results showed that MG132 treatment alone increased the SIRT2 protein level compared to the DMSO control group. Furthermore, when cells were pre-treated with MG132, DHA could no longer reduce the SIRT2 protein level. Consistent with this, the Co-IP results demonstrated that DHA increased the ubiquitination of SIRT2 (Fig. 6E). In addition, RT-qPCR analysis revealed that DHA treatment significantly decreased the mRNA expression level of IGFBP1, while the mRNA level of SIRT2 remained unchanged (Supplementary Figure S4).

Furthermore, western blotting was used to study the effects of DHA on the protein levels of SIRT2, IGFBP1, p-PI3K, PI3K, p-AKT, AKT, p-mTOR, and mTOR (Fig. 6F). A rescue experiment was also performed to explore the underlying molecular mechanism, which revealed that the DHA-mediated suppression of cell colony formation was partially reversed by overexpressing IGFBP1 (Fig. 6G) and that the DHA-mediated decreases in protein levels, including the levels of p-PI3K, p-AKT and p-mTOR, were also partially reversed via transfection of an IGFBP1-overexpressing plasmid (Fig. 6H). To examine the effect of SIRT2 degradation on its direct downstream targets, the acetylation levels of α-tubulin and NF-κB p65 were measured. As expected, the DHA-induced decrease in SIRT2 protein led to a significant accumulation of acetyl-α-tubulin (Lys40) and acetyl-NF-κB p65 (Lys310) (Fig. 6I). Collectively, these results indicate that DHA blocks SIRT2-induced signal transduction by promoting the ubiquitin–proteasome degradation of SIRT2, subsequently inhibiting IGFBP1 transcription.

## Discussion

As the only cytoplasm-localized member of the SIRT family, SIRT2 is becoming increasingly attractive because of its multiple roles in regulating physiological and pathological processes in humans, especially in the tumorigenesis of different types of cancer; however, the roles of SIRT2 are controversial because it has both tumour-promoting and tumour-suppressing functions^5,24^. Early studies reported that SIRT2 is distributed mainly in the cytoplasm, although small amounts of SIRT2 can be detected in both the mitochondria and the nucleus^5^. In this study, we thoroughly investigated the distribution pattern and expression level of SIRT2 both in liver cancer tissues and in liver cancer cells and showed that SIRT2 is highly expressed in liver cancer tissues and distributed in the cytoplasm of liver cancer cells (Fig. 1). Furthermore, the functional role of SIRT2 in HCC appears to be heavily dependent on post-translational regulation, such as ubiquitin-dependent degradation, rather than mere mRNA transcript abundance. Early studies have shown that during the development of liver cancer, SIRT2 can mediate epithelial-to-mesenchymal transition^25^, invasion^26^, multidrug resistance^2^, and immune evasion^2^. In this study, further functional research was performed to confirm the role of SIRT2 in the development of liver cancer and revealed that the expression level of SIRT2 is positively related to cell proliferation activity and tumour growth both in vitro and in vivo (Figs. 2, 3). These results further support that SIRT2 is a promising oncogenic target and exhibits tumour-promoting activity in the cytoplasm of liver cancer cells.

Although our findings demonstrate an oncogenic role of SIRT2 in advanced hepatocellular carcinoma, it is important to acknowledge that the biological functions of SIRT2 are highly context-dependent, and it exerts significant protective effects in non-cancer disease models and other malignancies. For instance, a recent study elegantly revealed that in non-cancerous hepatocytes, SIRT2 plays a crucial hepatoprotective role against lipotoxicity; palmitic acid induces hepatocyte inflammation by suppressing the BMAL1-NAD^+^-SIRT2 axis, indicating that physiological levels of SIRT2 are required to maintain metabolic homeostasis for liver^27^. Furthermore, in breast cancer, SIRT2 frequently acts as a tumor suppressor during early carcinogenesis. It has been reported to maintain genomic stability and restrict the antioxidant activity of specific proteins, thereby sensitizing early-stage breast cancer cells to reactive oxygen species (ROS)-induced DNA damage and apoptosis. This dual nature suggests that while pharmacological inhibition of SIRT2 by agents like DHA offers a promising strategy for halting progressive liver cancer via the IGFBP1/PI3K/AKT/mTOR axis, the systemic effects of SIRT2 modulation on healthy tissue homeostasis and its varying roles across different cancer types warrant careful consideration in future clinical applications.

The liver plays an important role in regulating insulin-mediated metabolism, and IGFBP1, a key component of the IGF system, is receiving increasing attention in the development of cancer. IGFBP1 has been reported to regulate tumour differentiation, cell invasion, metastasis, survival, and proliferation^28–30^ and to contribute to drug resistance^10^. However, not only is the expression pattern and function of IGFBP1 in tumorigenesis under debate^4,9,31^, but the association between IGFBP1 expression and cancer risk is also controversial^32^ and needs further study. In this study, we found that IGFBP1 can promote liver cancer cell proliferation and colony formation by activating the PI3K/AKT/mTOR signalling pathway (Figs. 4, 5, 6). SIRT2 can activate the PI3K/AKT/mTOR signalling pathway by upregulating IGFBP1 expression, and that knockdown of IGFBP1 expression can impair SIRT2 expression-induced cell proliferation and signal transduction (Fig. 4). These studies indicate that IGFBP1 is an effective downstream target through which SIRT2 exerts its oncogenic activity. Although our proteomic data showed that SLC2A1 (GLUT1) had a higher fold-change, it is a universal glucose transporter commonly upregulated in solid tumors. We focused on IGFBP1 in this study because it acts as a specific signalling molecule that connects SIRT2 to the PI3K/AKT/mTOR pathway.

The PI3K/Akt/mTOR signalling pathway plays crucial roles in the process of tumorigenesis and is one of the most commonly dysregulated signalling pathways in cancer ^33^. Treatment with inhibitors of the PI3K/AKT/mTOR pathway, such as everolimus, has been shown to improve clinical outcomes in certain malignancies like breast cancer^34^. In advanced hepatocellular carcinoma, multikinase inhibitors that suppress this signalling axis, including lenvatinib and regorafenib, have been shown to significantly improve overall survival in phase III clinical trials^35,36^. Furthermore, specific PI3K and AKT inhibitors are currently under active clinical investigation for liver cancer therapy^37^. Exploring low-toxicity, high-efficiency inhibitors of the PI3K/Akt/mTOR signalling pathway to improve outcomes is a precision approach for cancer therapy. DHA is well known not only for its low toxicity and potent antitumour activity but also for its reported synergistic anticancer effects with a variety of drugs used in clinical therapy^20,38,39^. However, the molecular mechanism by which DHA inhibits the tumorigenesis of liver cancer is still obscure. In this study, we revealed that DHA downregulates SIRT2 expression in a ubiquitination‐dependent manner and then impairs SIRT2-IGFBP1-induced PI3K/Akt/mTOR signalling pathway activation; moreover, during this process, IGFBP1 is crucial for the activation of PI3K/Akt/mTOR signal transduction mediated by SIRT2 and that DHA exhibits antitumour activity (Fig. 6).

While exploring clinical databases, we noted that the mRNA levels of SIRT2 and IGFBP1 in bulk RNA-seq datasets (such as TCGA) do not fully align with their protein-level functions in HCC. Because SIRT2 is strongly regulated by post-translational ubiquitin-dependent degradation, its mRNA abundance cannot accurately represent the functional protein level. Furthermore, normal hepatocytes secrete high levels of IGFBP1 for systemic metabolic regulation. The dedifferentiation of HCC cells leads to a loss of this endocrine function, which masks the local autocrine/paracrine oncogenic effects of IGFBP1 in bulk tissue analysis. Therefore, evaluating their clinical relevance relies more on protein-level assessments rather than transcriptomic data.

In this study, we have showed that SIRT2 is an effective target and DHA is a promising drug in liver cancer therapy for its low toxicity and high antitumour activity. However, there were still some limitations of the research results: the role of SIRT2-IGFBP1-induced signalling pathway in liver cancer metastasis is still need to further study, and the molecular mechanism of SIRT2 in regulating IGFBP1 in detail is still unknown. We will further study the regulating mechanism and function of SIRT2 in the development of liver cancer and exploring the application value in the later study.

## Conclusion

**Fig. 7:**
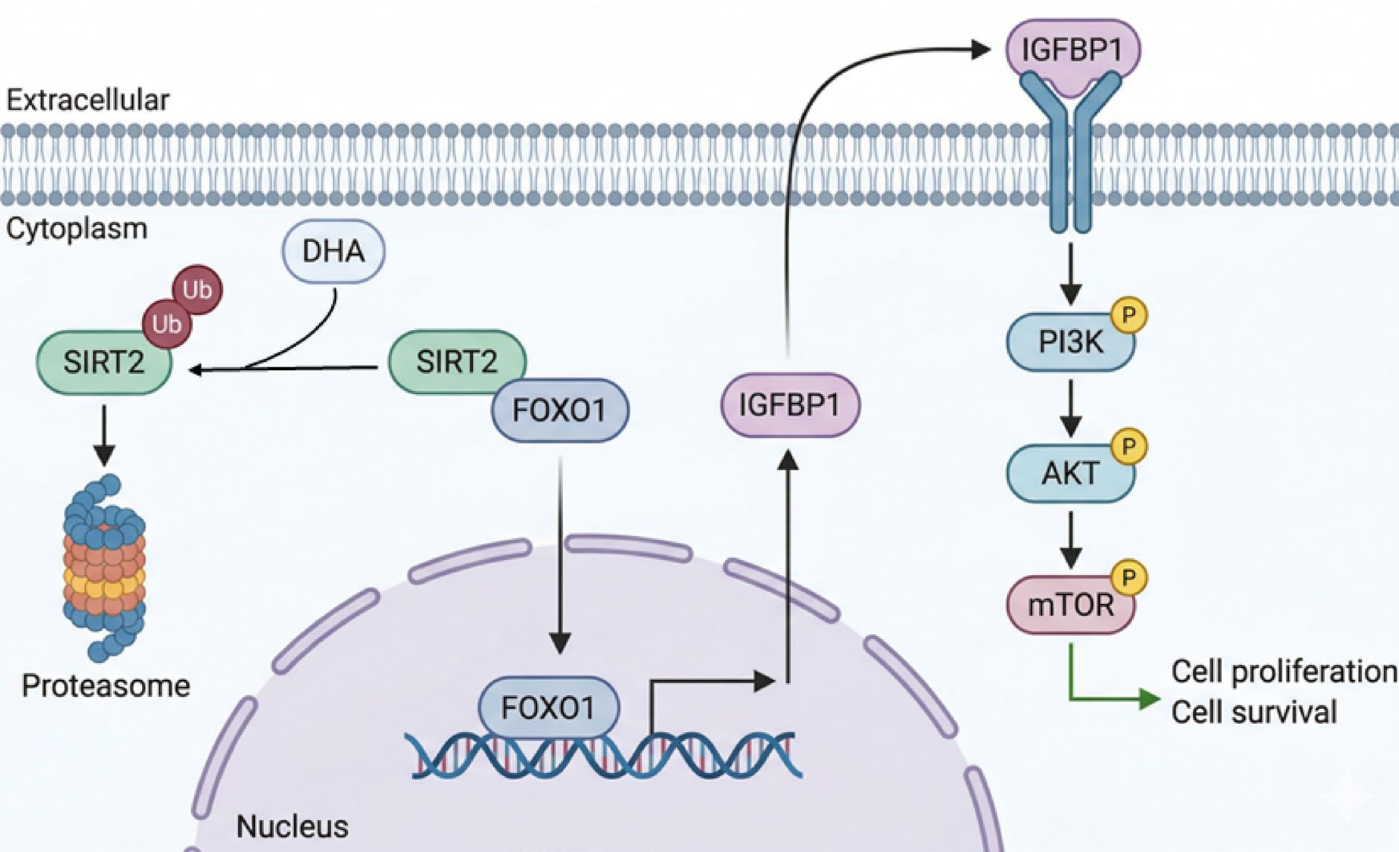
Schematic diagram of the molecular mechanism. DHA promotes the ubiquitin–proteasome degradation of SIRT2, which suppresses the SIRT2-mediated transcriptional upregulation of IGFBP1. The reduction of IGFBP1 secretion blocks the activation of the downstream PI3K/AKT/mTOR signalling pathway, ultimately inhibiting liver cancer cell proliferation and survival.

SIRT2 is functioning as an oncogene in liver cancer cells and exerts its protumour effects by upregulating IGFBP1 expression and subsequently activating PI3K/Akt/mTOR signal transduction. We found that DHA can block IGFBP1-induced PI3K/Akt/mTOR signal transduction by suppressing SIRT2 expression in a ubiquitination‐dependent manner. In addition, IGFBP1 is found to be crucial for SIRT2-mediated activation of PI3K/Akt/mTOR signal transduction and for the antitumour activity of DHA (Fig. 7).

## Supplementary Information

Supplementary Material 1.

Supplementary Material 2.

Supplementary Material 3.

## Data Availability

The processed data that support the findings of this study are included in this published article and its supplementary information files. The raw mass spectrometry datasets generated and analysed during the current study are not publicly available because they are part of an ongoing project currently being utilized by other group members for separate studies, but are available from the corresponding author on reasonable request.
