# Supplementary material for: Dihydroartemisinin exerts antitumour activity by blocking SIRT2-IGFBP1-induced PI3K/AKT/mTOR signal transduction in liver cancer
Source: Sci Rep. 2026 Apr 20;16:18299. doi: 10.1038/s41598-026-49240-5 (PMC13260731; doi:10.1038/s41598-026-49240-5)

Original data of Figure 1

Figure 1B SIRT2

1

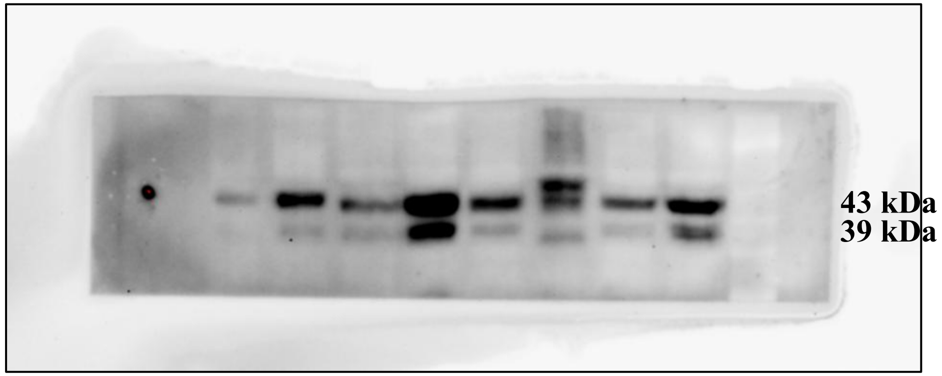

2

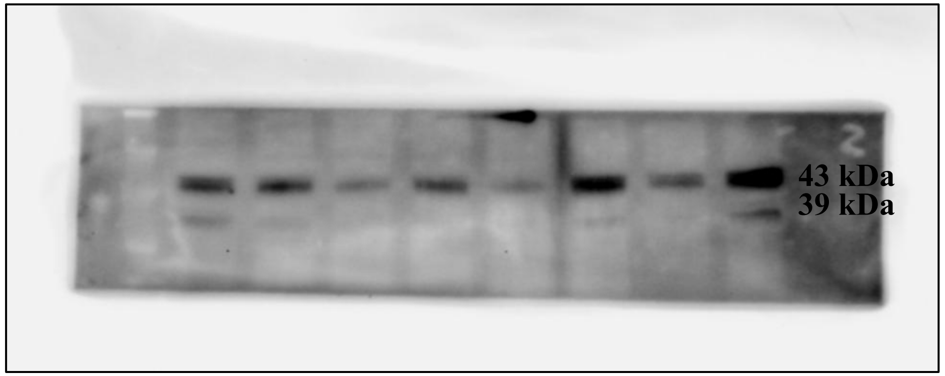

Figure 1B GAPDH

1

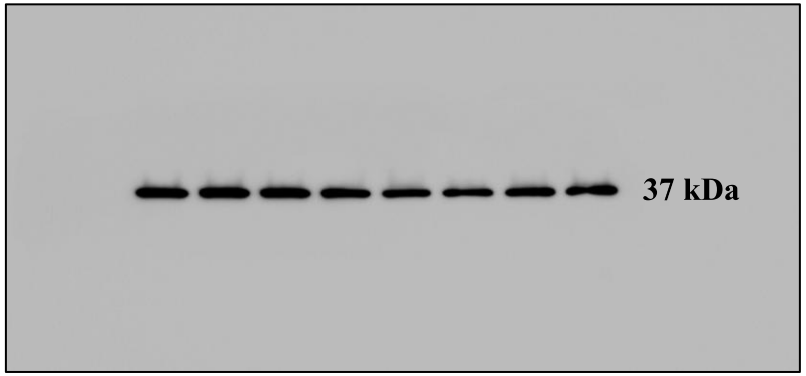

2

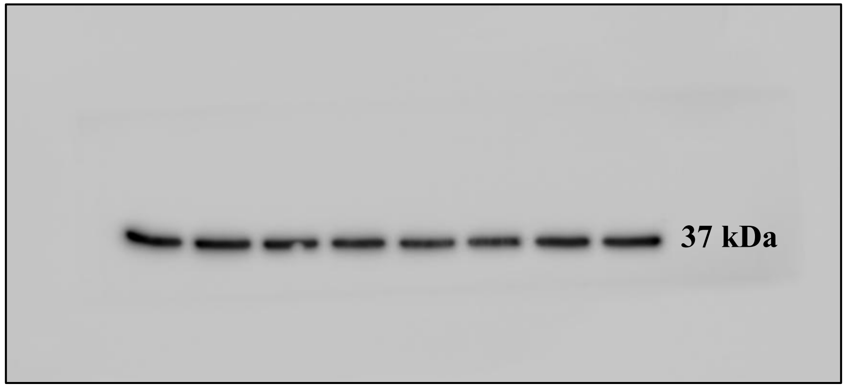

Original data of Figure 1

Huh7

Figure 1D SIRT2

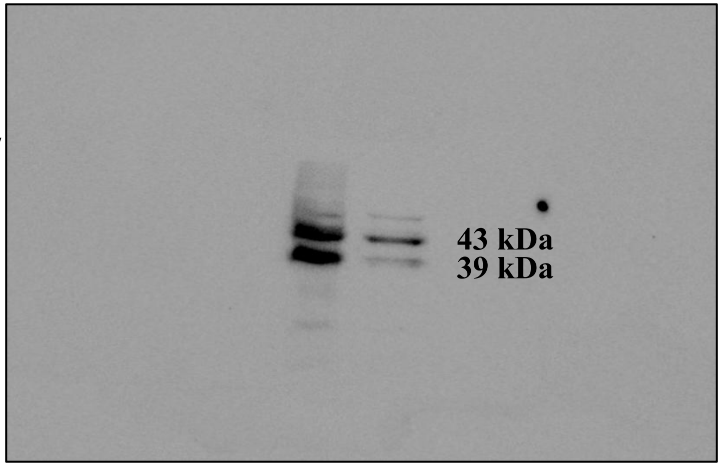

Figure 1D Histone H3

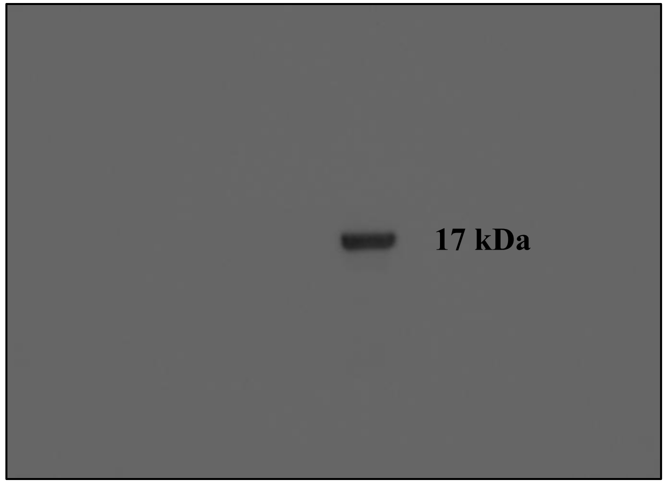

Figure 1D GAPDH

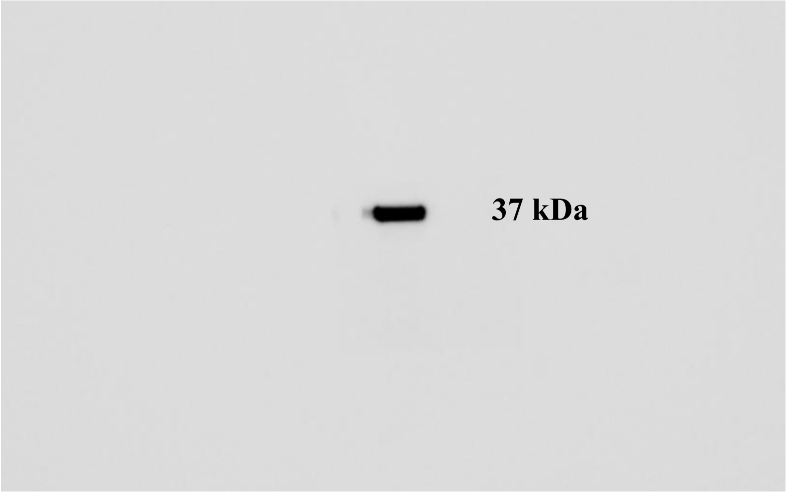

HepG2

Figure 1D SIRT2

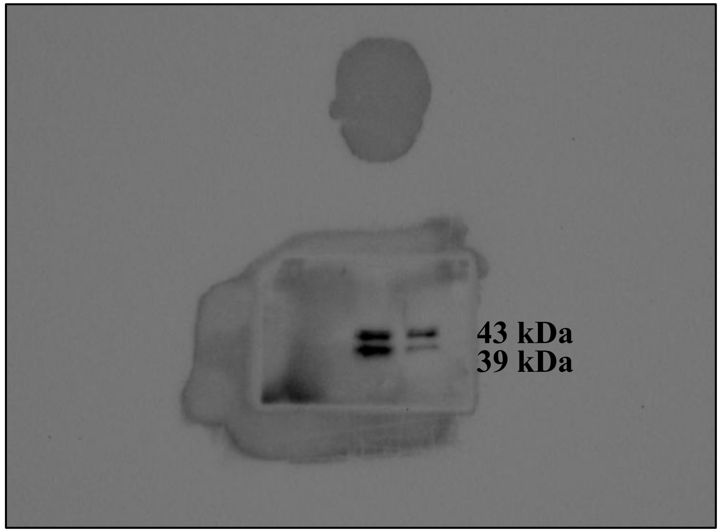

Figure 1D Histone H3

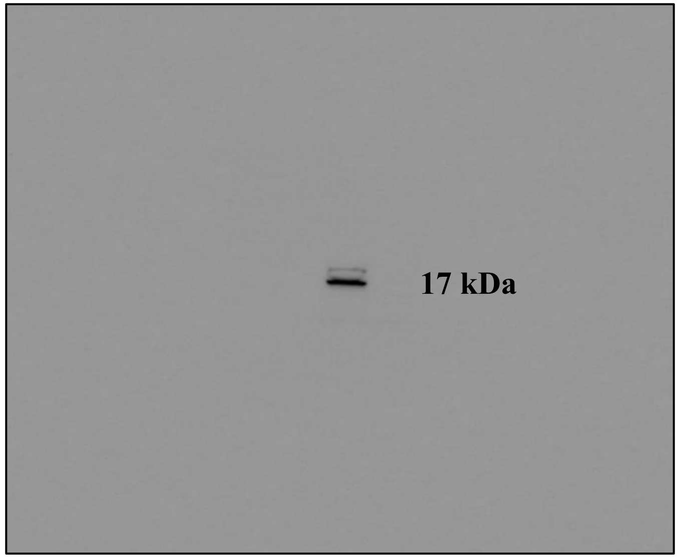

Figure 1D GAPDH

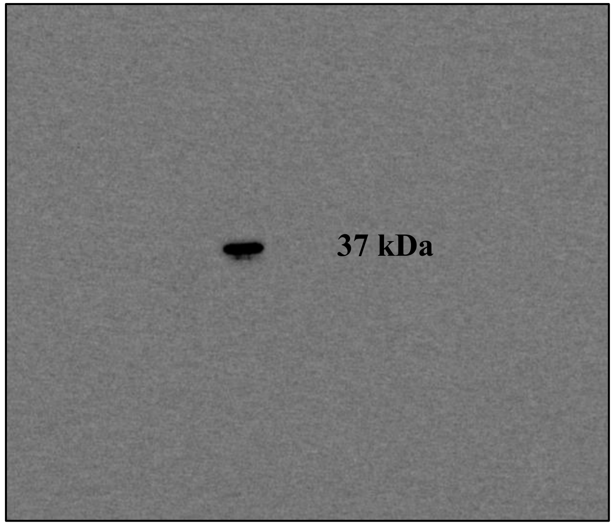

Original data of Figure 2

Figure 2A FLAG

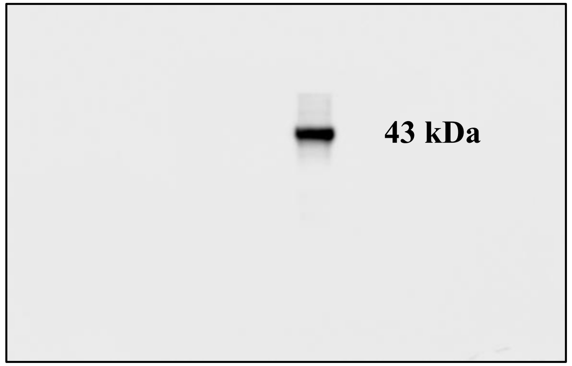

Figure 2A SIRT2

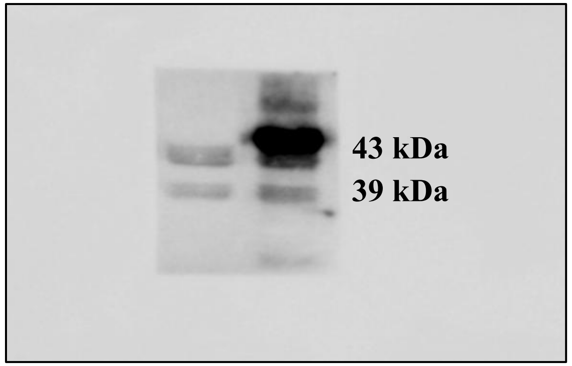

Figure 2A GAPDH

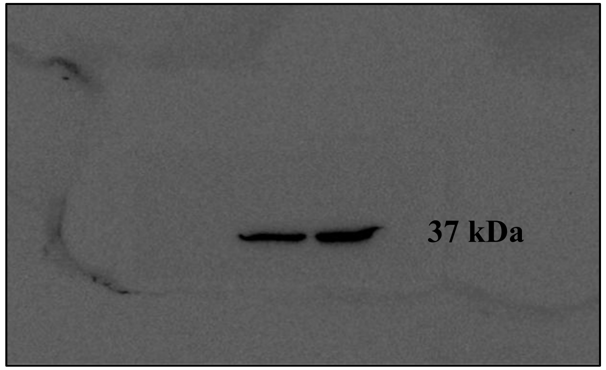

Figure 2B FLAG

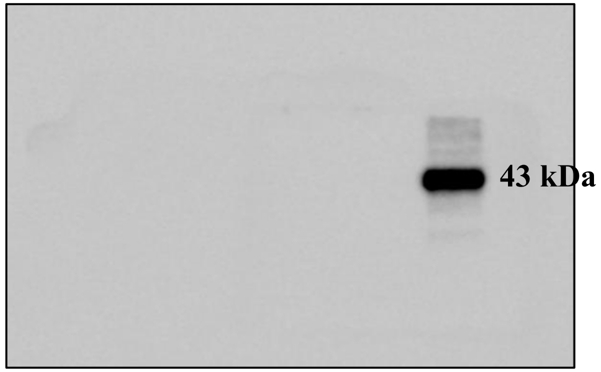

Figure 2B SIRT2

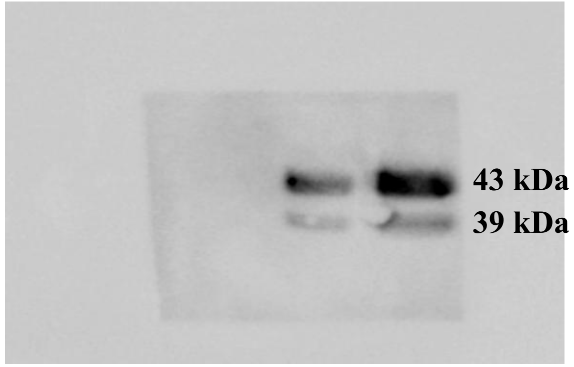

Figure 2B GAPDH

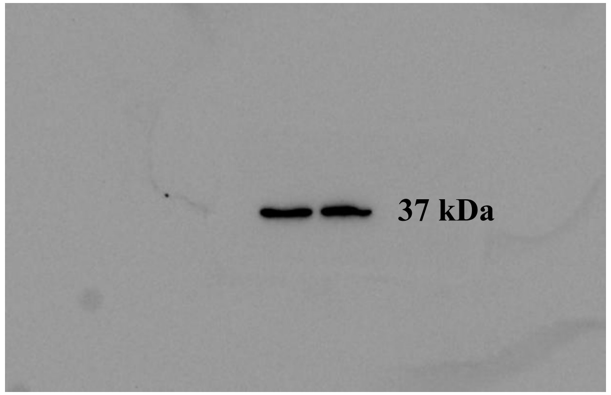

Original data of Figure 2

Figure 2G SIRT2

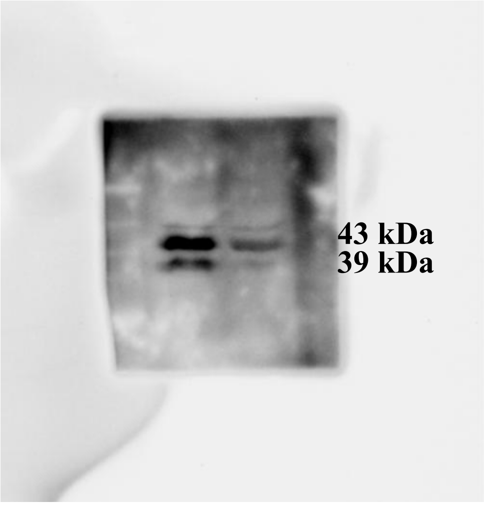

Figure 2H SIRT2

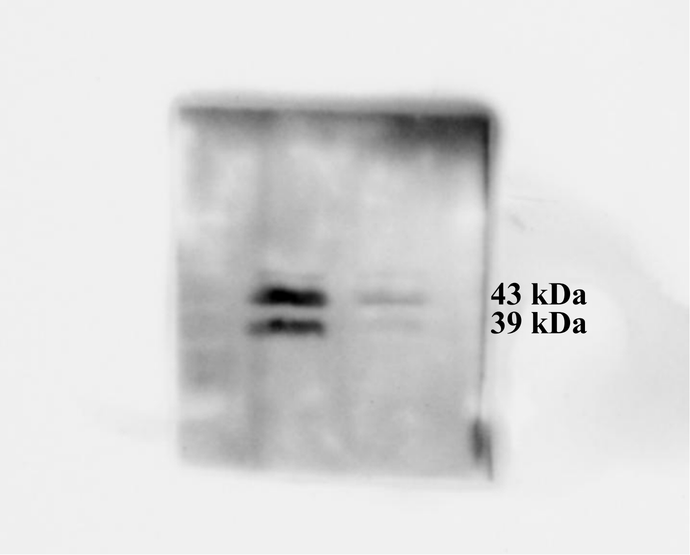

Figure 2G GAPDH

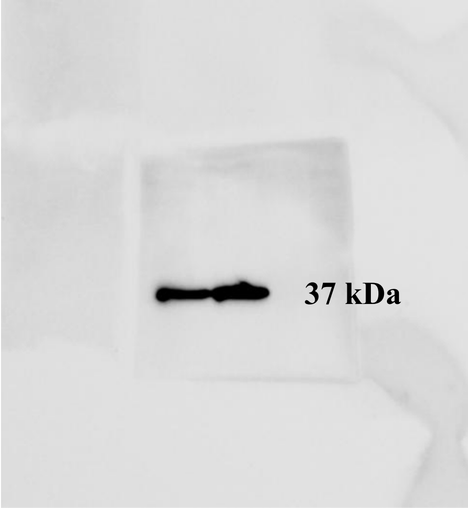

Figure 2H GAPDH

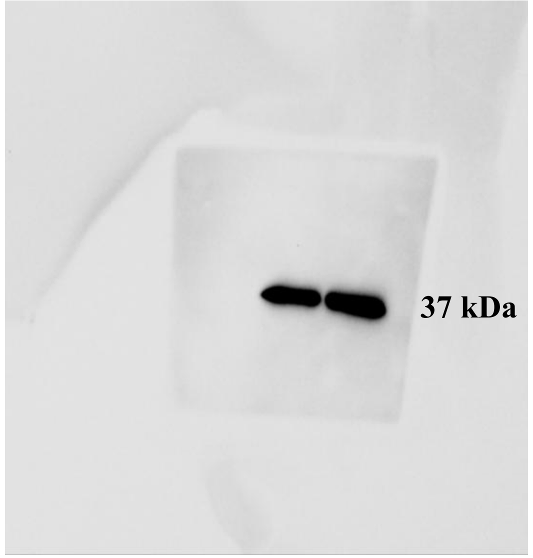

Original data of Figure 3

Figure 3B FLAG

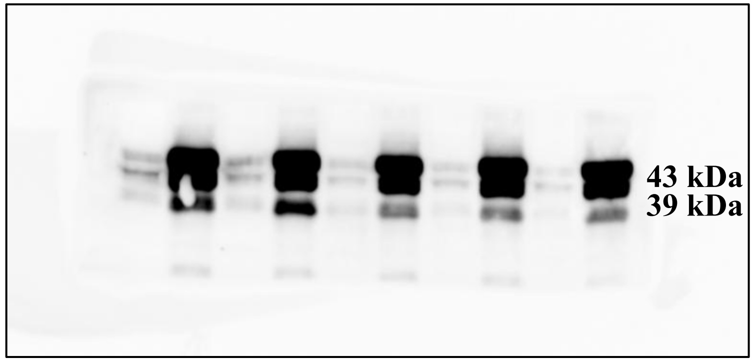

Figure 3B GAPDH

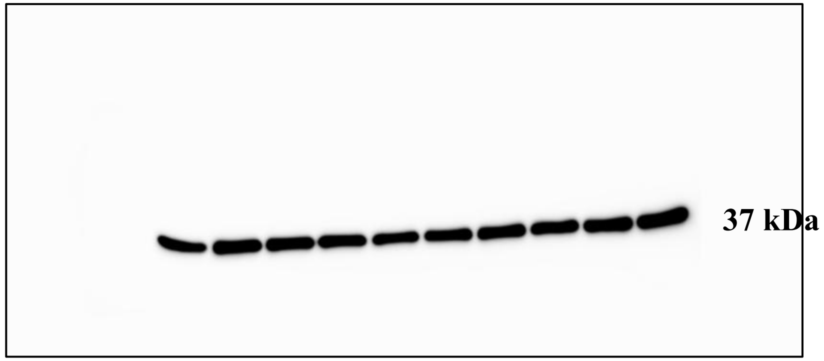

Figure 3D SIRT2

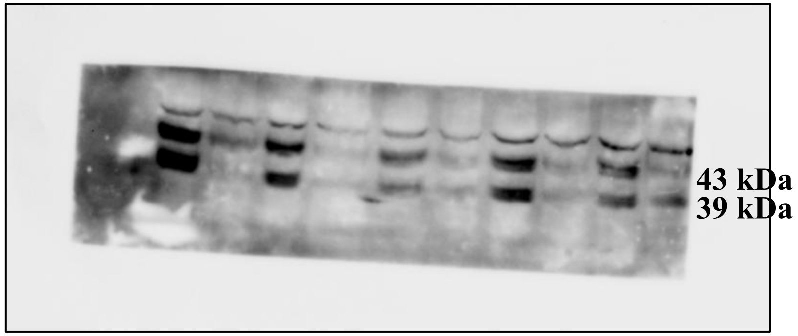

Figure 3D GAPDH

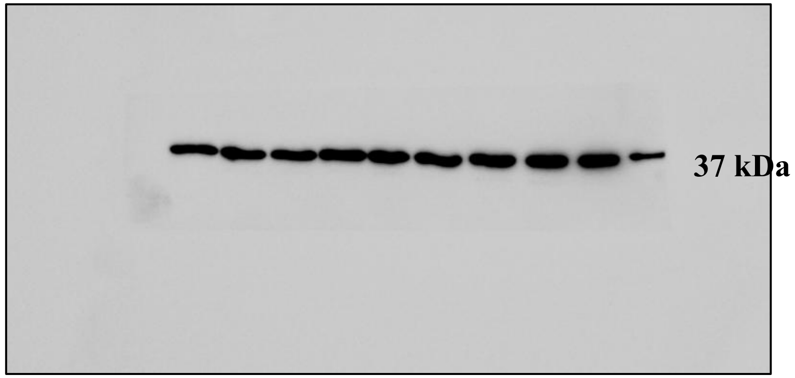

Original data of Figure 4

Figure 4A IGFBP1

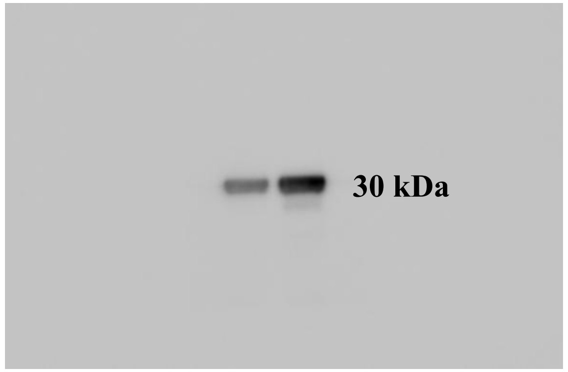

Figure 4A FLAG

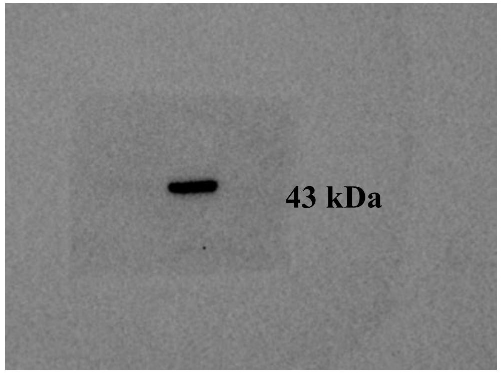

Figure 4A GAPDH

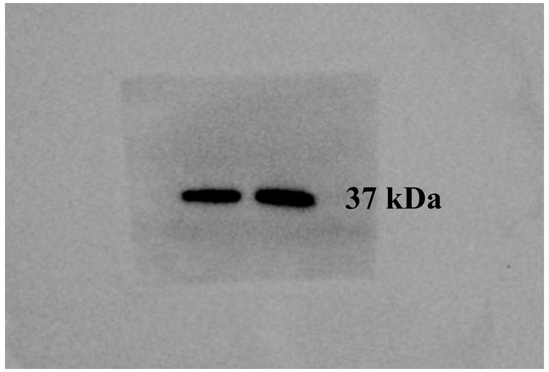

Figure 4A IGFBP1

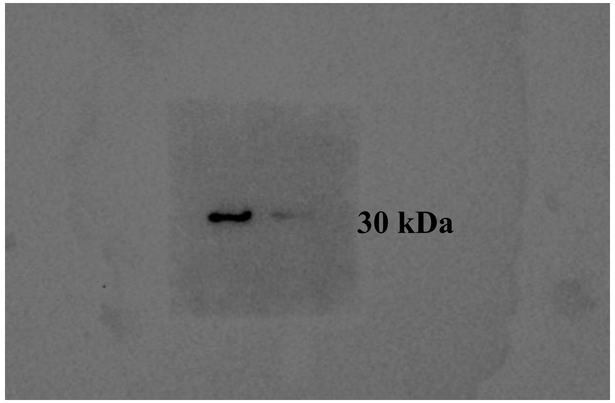

Figure 4A SIRT2

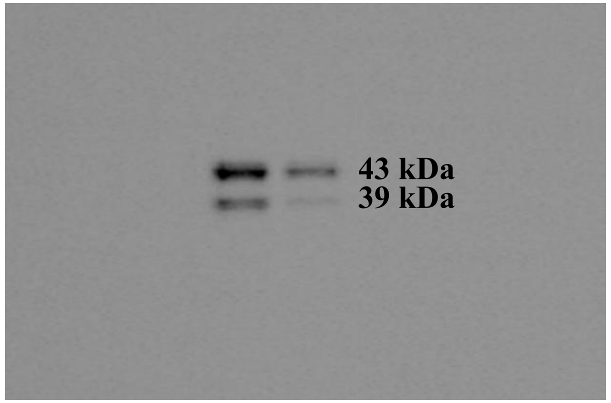

Figure 4A GAPDH

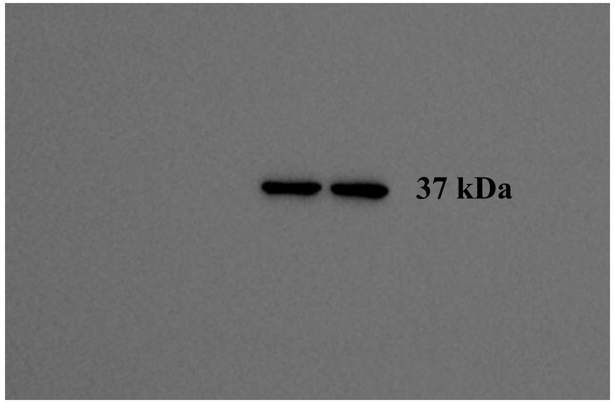

Original data of Figure 4

Figure 4D p-mTOR

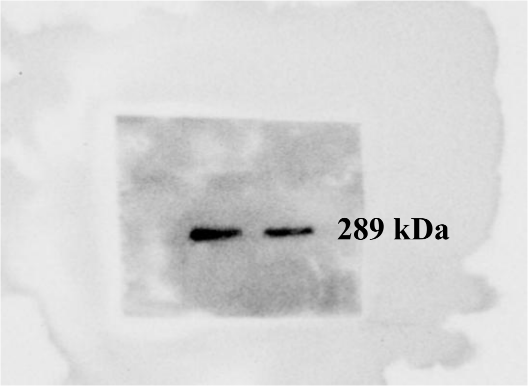

Figure 4D mTOR

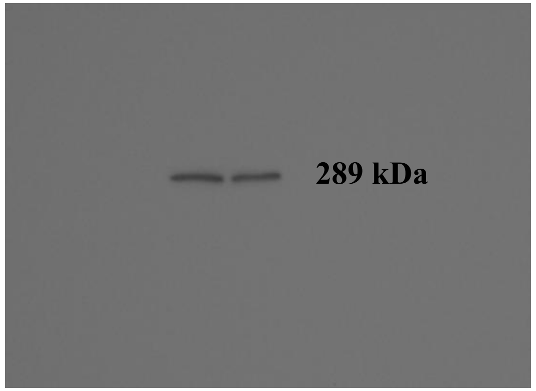

Figure 4D p-AKT

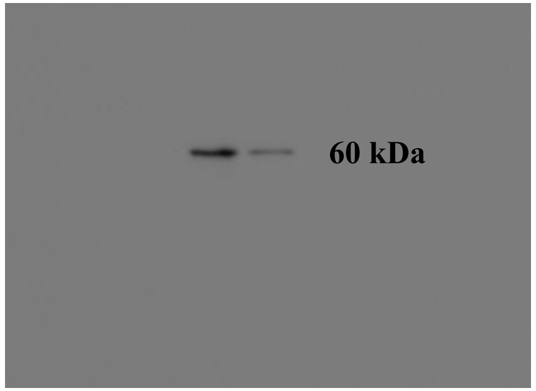

Figure 4D AKT

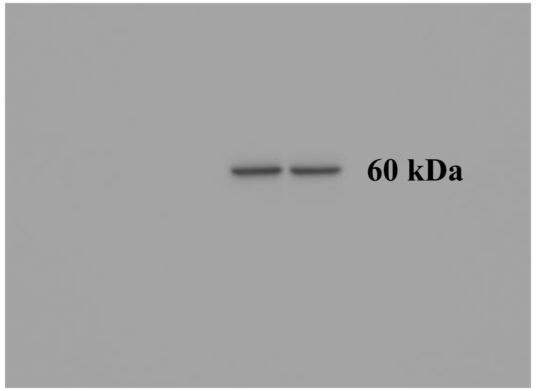

Figure 4D p-PI3K

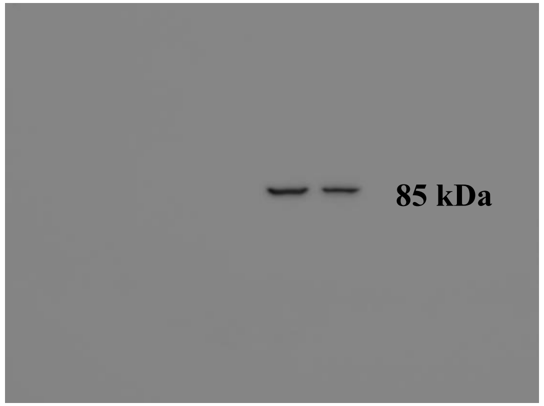

Figure 4D PI3K

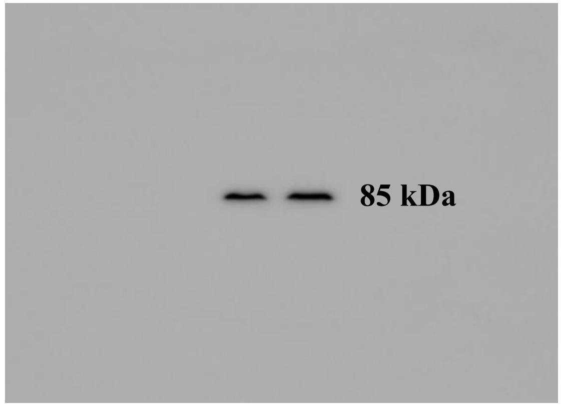

Figure 4D IGFBP1

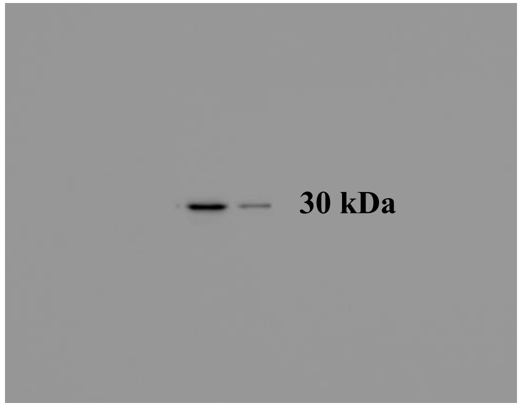

Figure 4D GAPDH

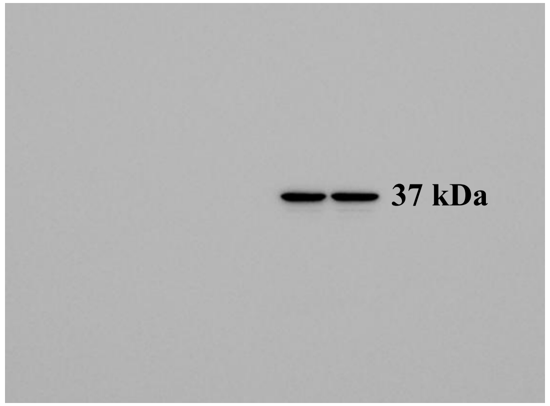

Original data of Figure 5

Figure 5A p-mTOR

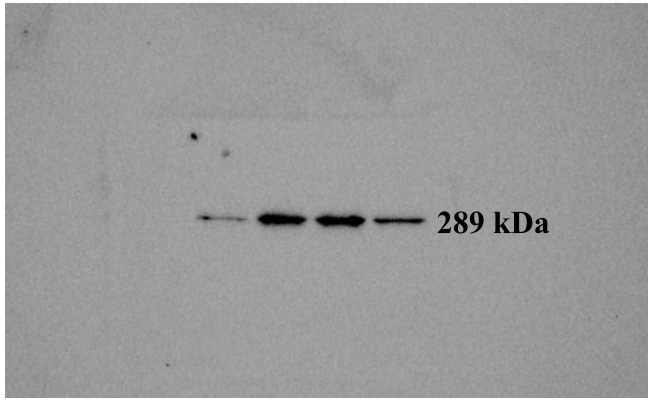

Figure 5A p-AKT

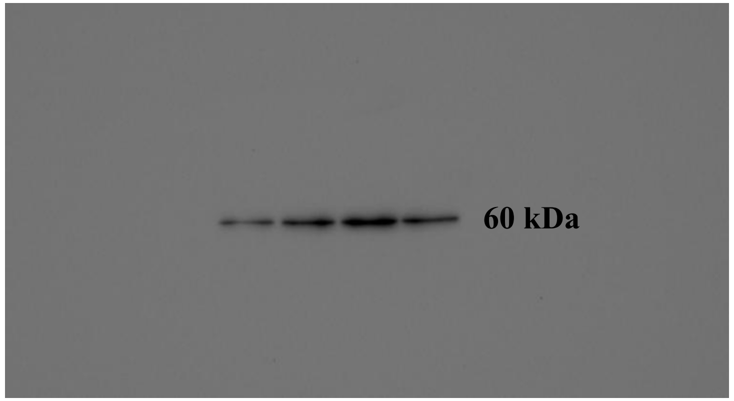

Figure 5A p-PI3K

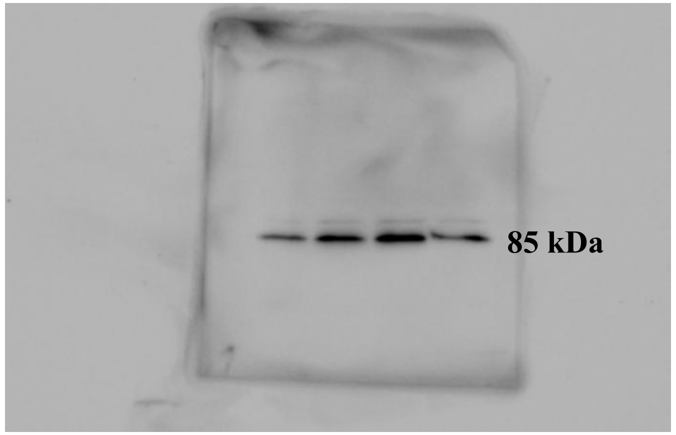

Figure 5A mTOR

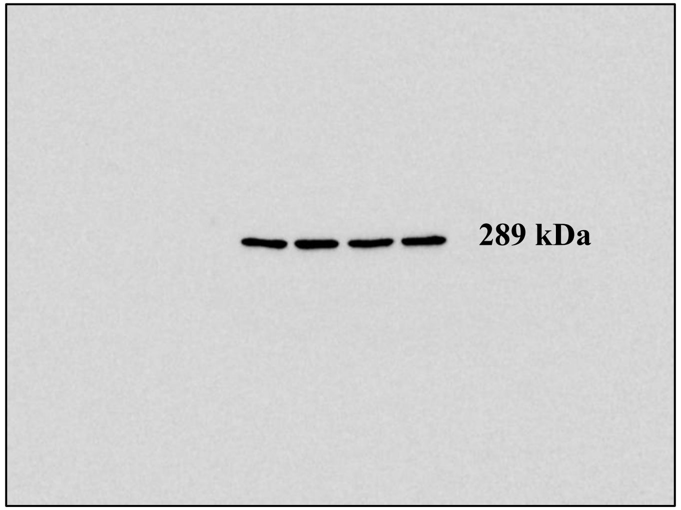

Figure 5A AKT

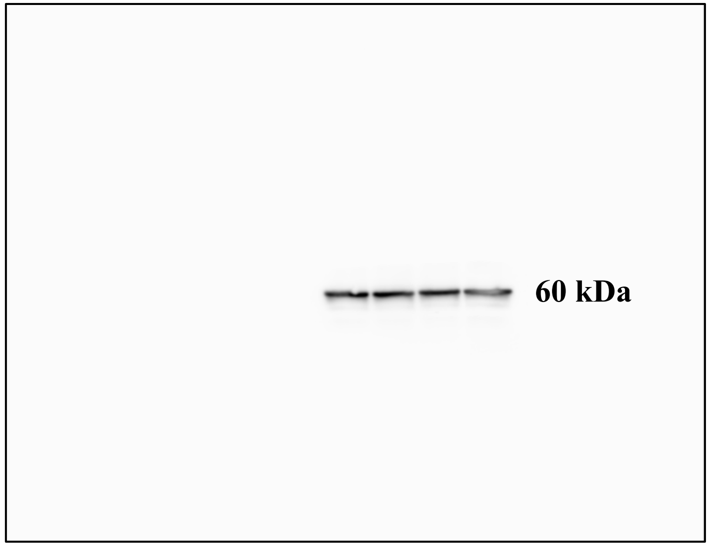

Figure 5A PI3K

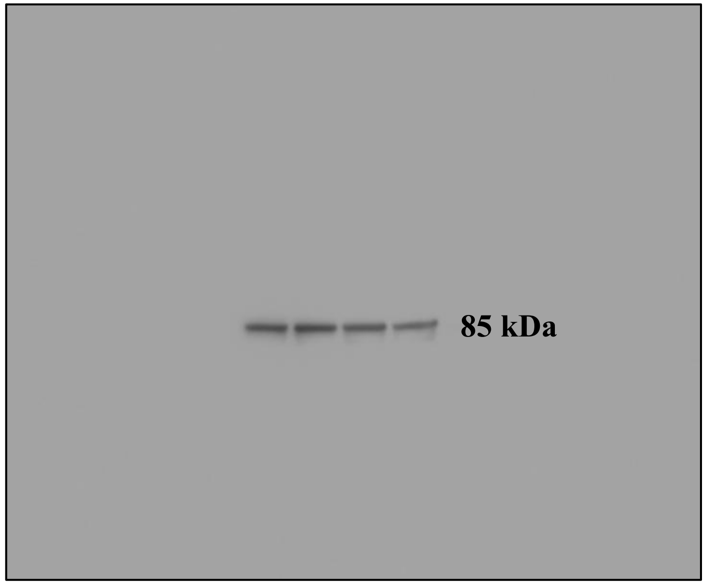

Original data of Figure 5

Figure 5A IGFBP1

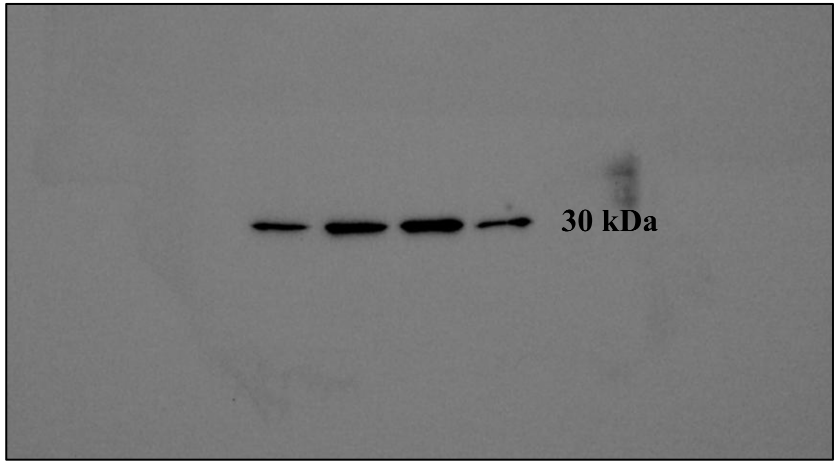

Figure 5A FLAG

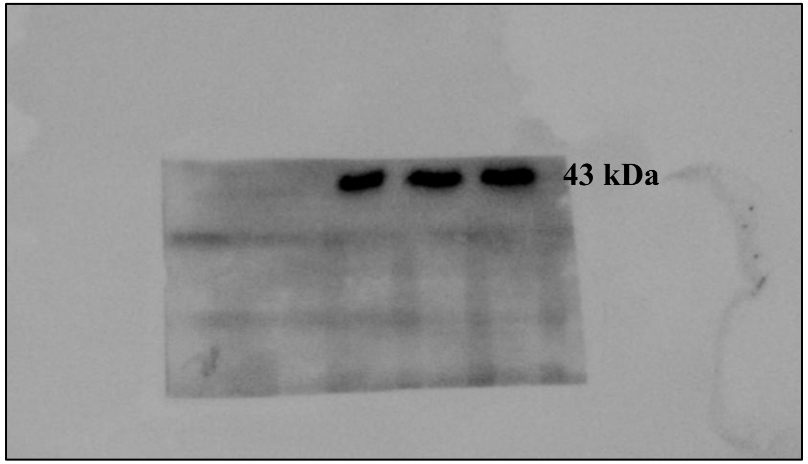

Figure 5A GAPDH

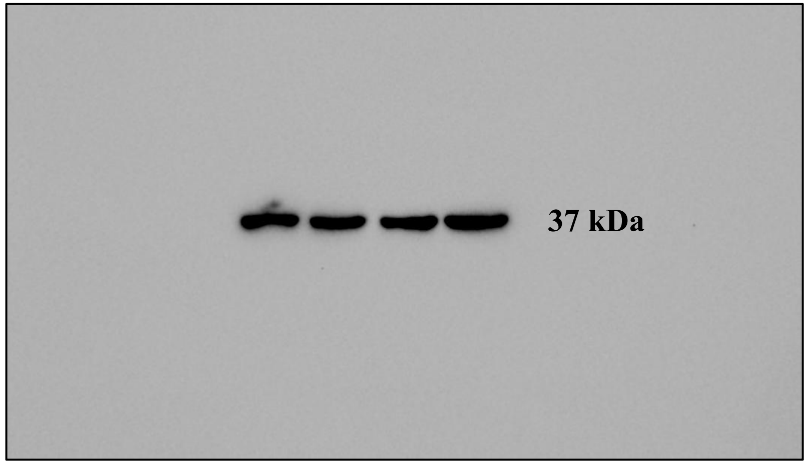

Original data of Figure 6

Figure 6D p-mTOR

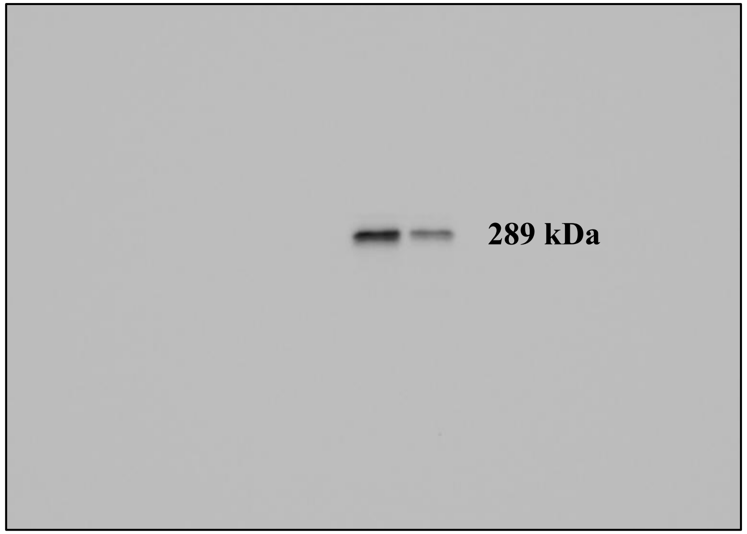

Figure 6D p-AKT

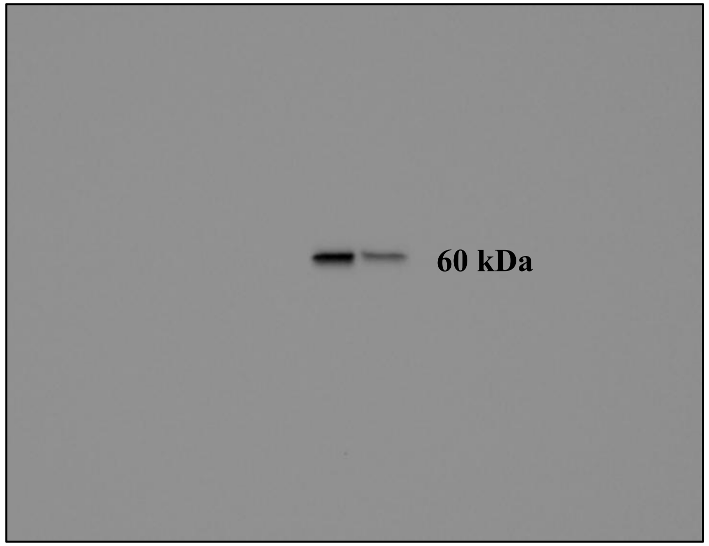

Figure 6D p-PI3K

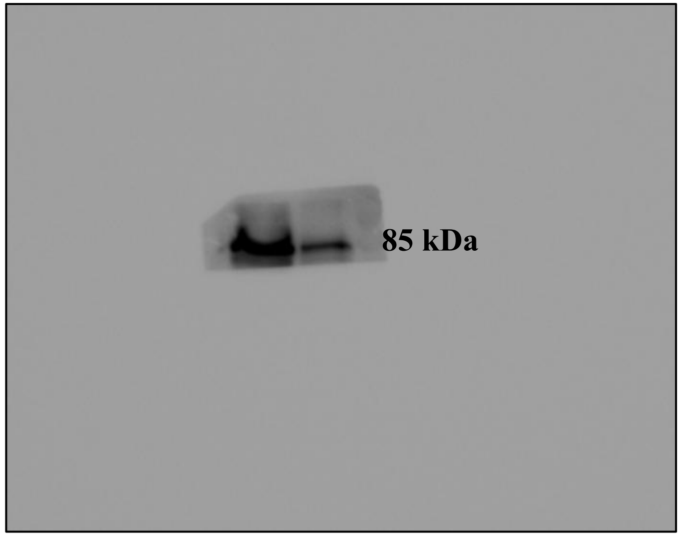

Figure 6D mTOR

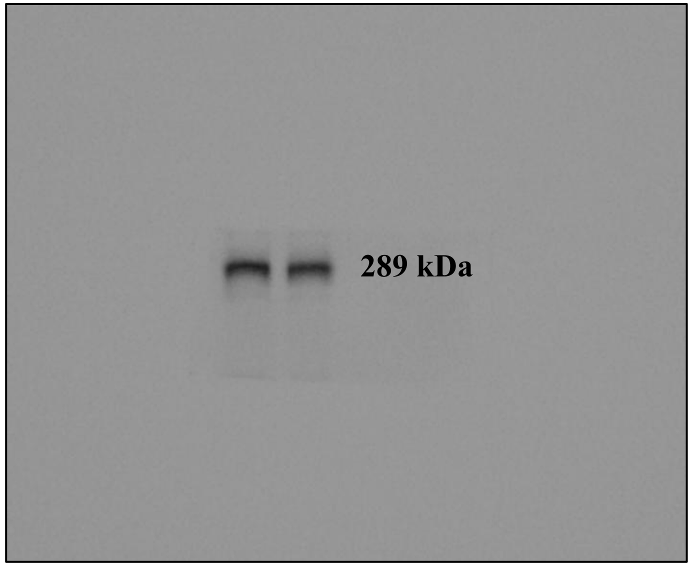

Figure 6D AKT

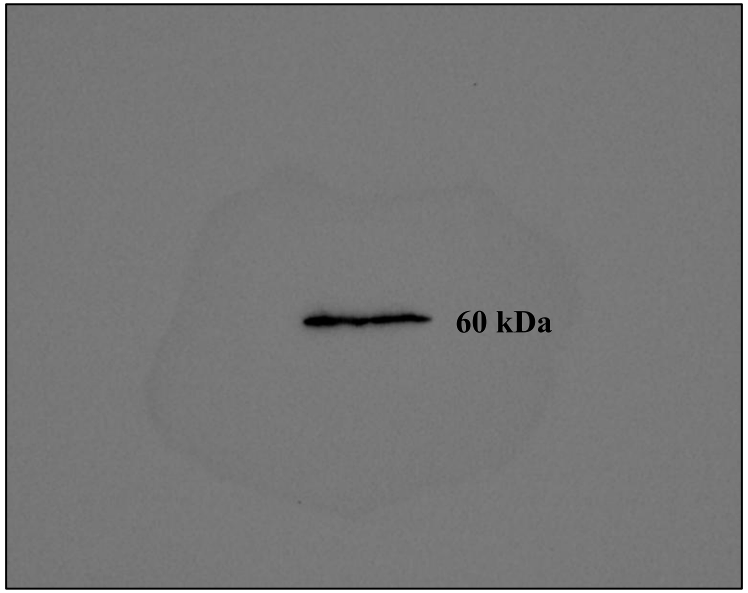

Figure 6D PI3K

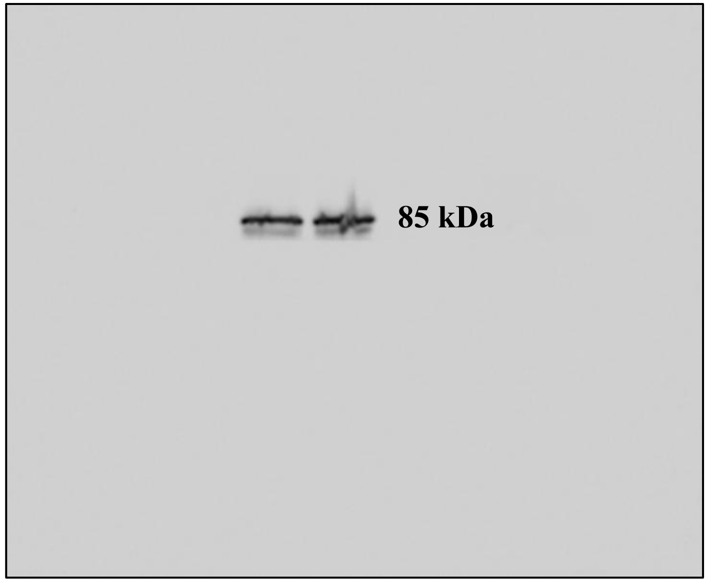

Original data of Figure 6

Figure 6D Cleaved Caspase 3

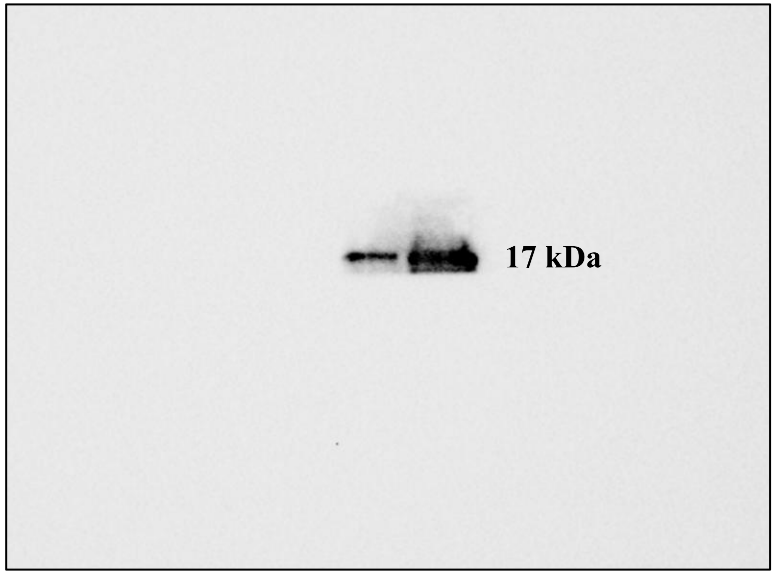

Figure 6D Caspase 3

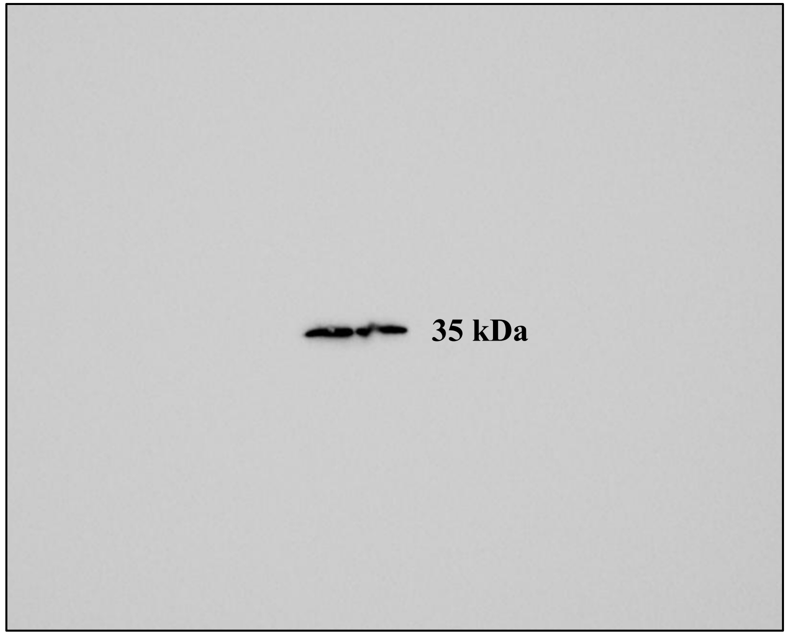

Figure 6D PCNA

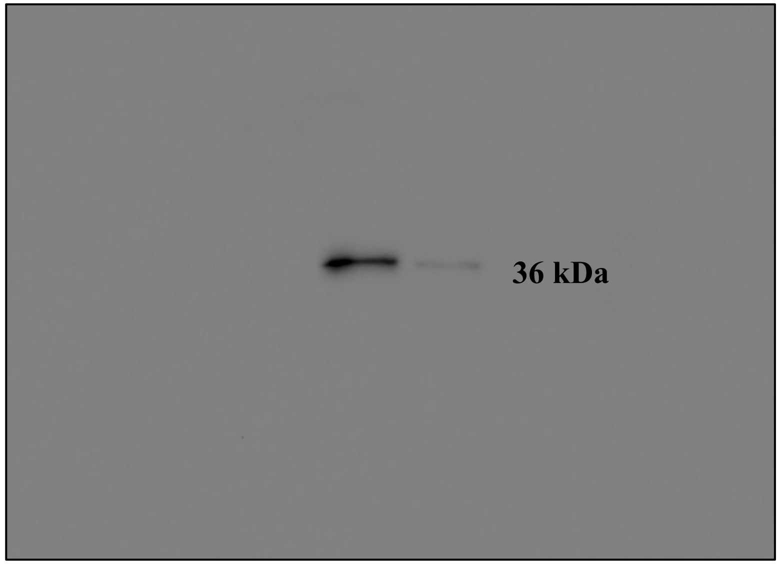

Figure 6D SIRT2

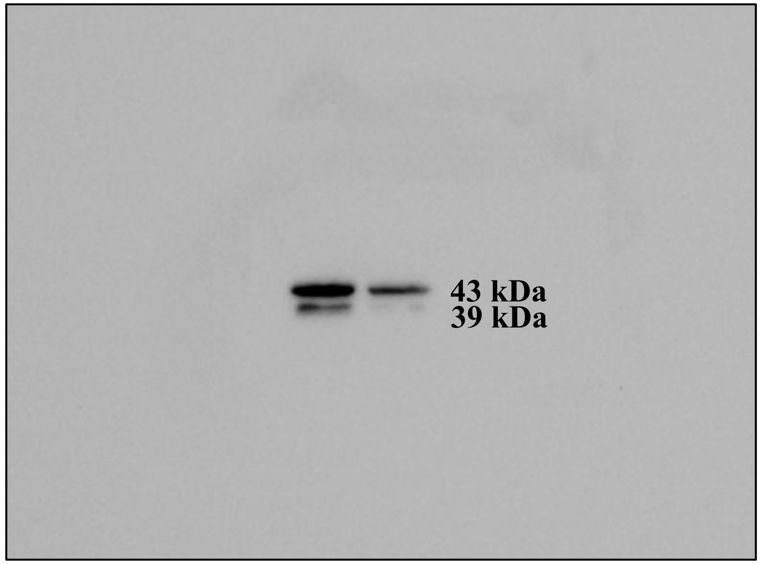

Figure 6D GAPDH

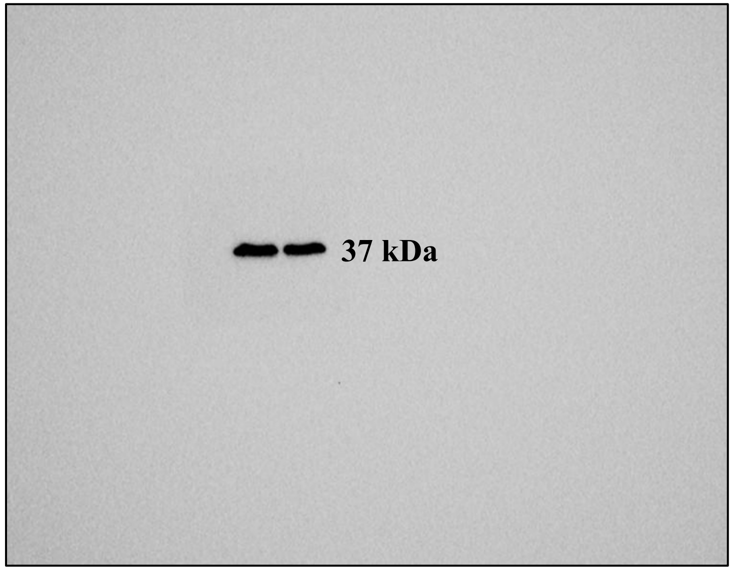

Original data of Figure 6

Figure 6E SIRT2

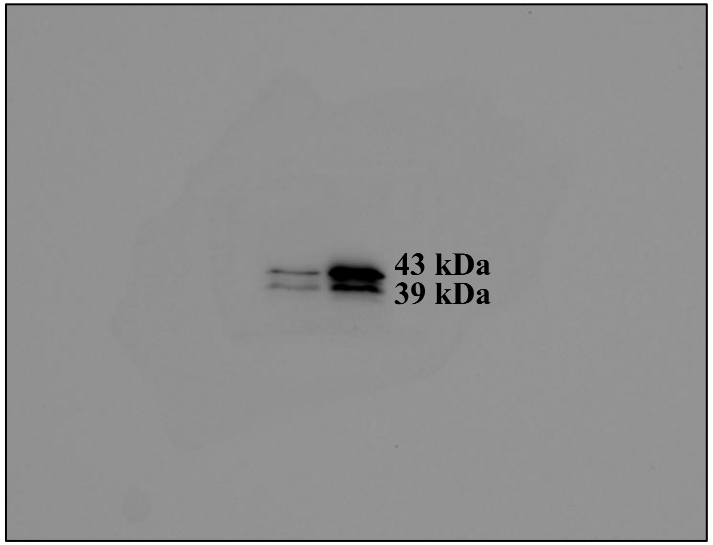

Figure 6E GAPDH

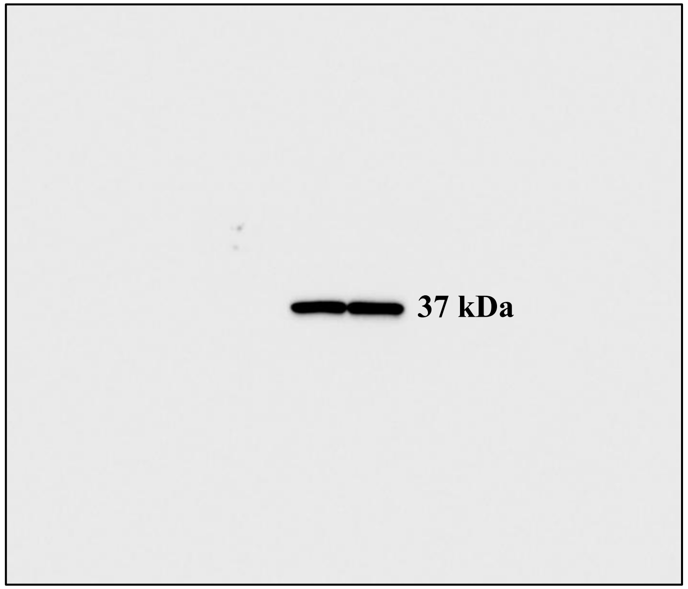

Figure 6E SIRT2

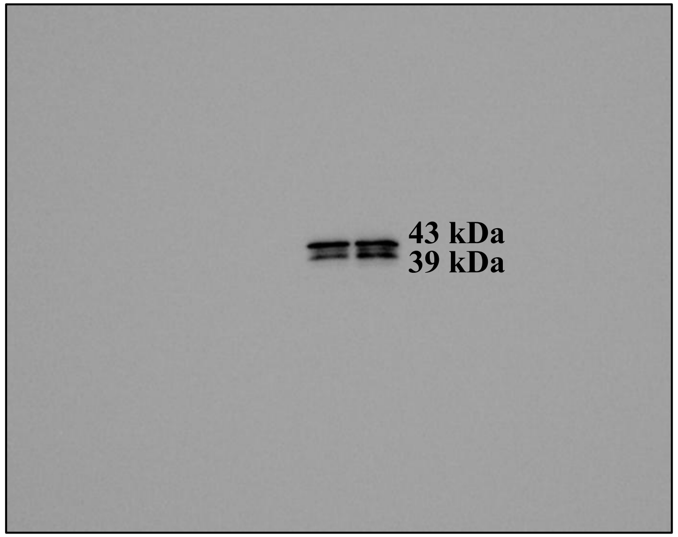

Figure 6E GAPDH

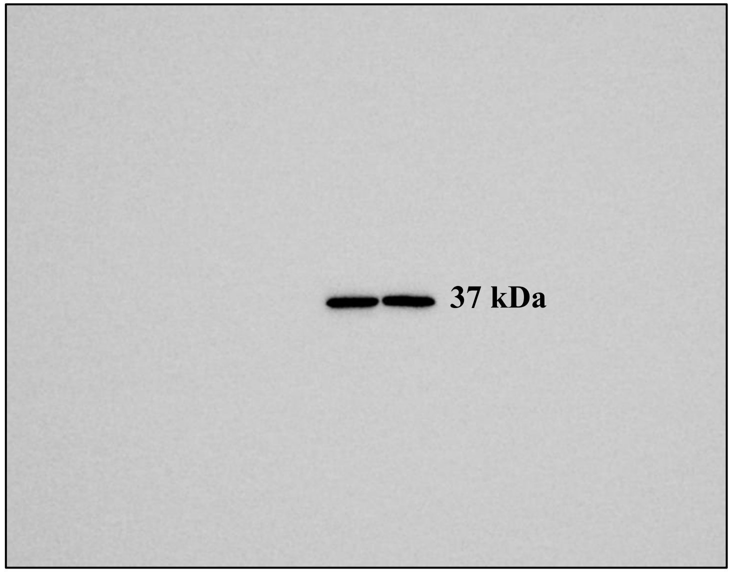

Original data of Figure 6

Figure 6E HA

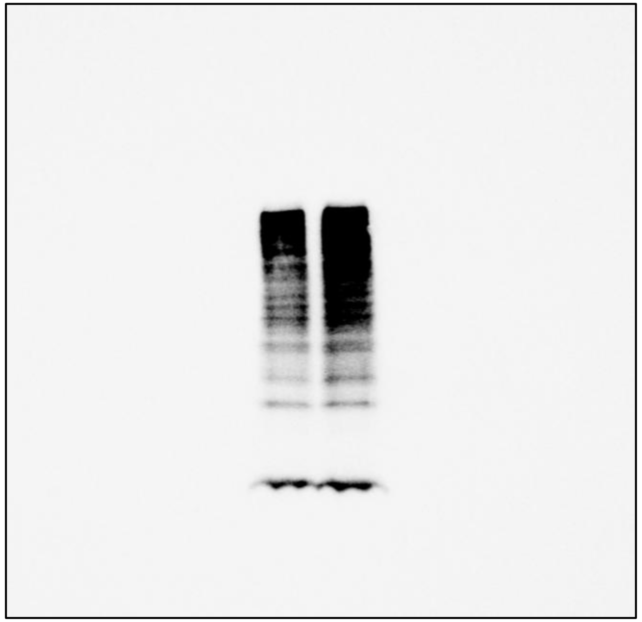

Figure 6E SIRT2

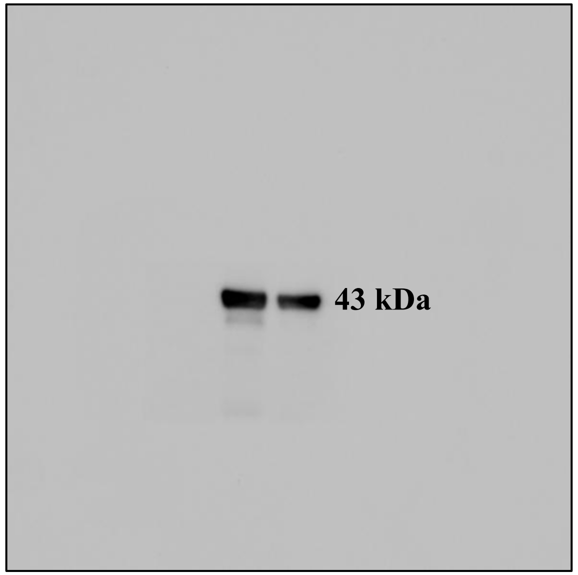

Figure 6E SIRT2

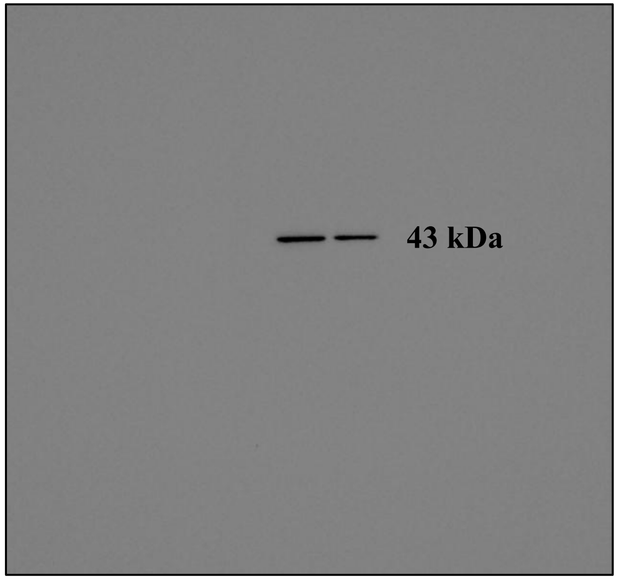

Figure 6E GAPDH

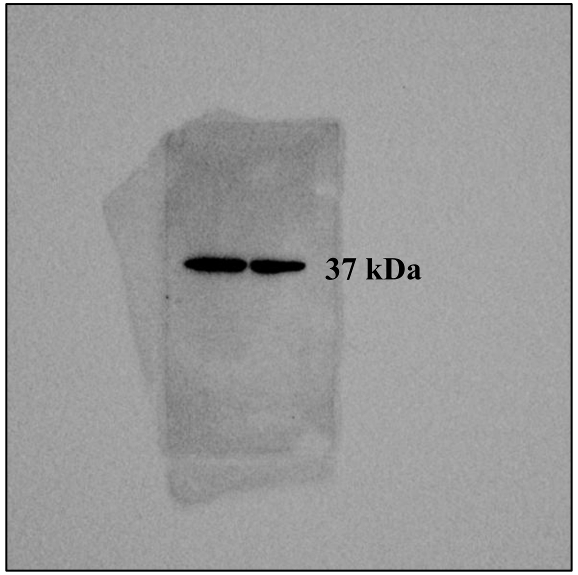

Original data of Figure 6

Figure 6F p-mTOR

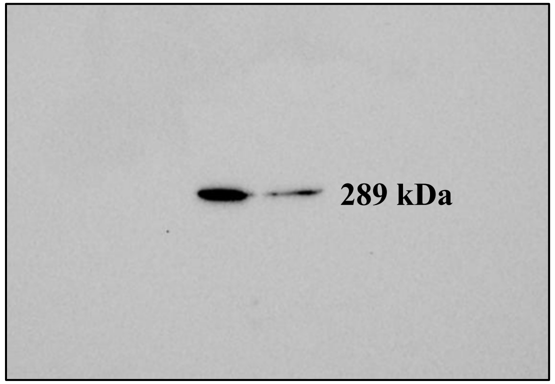

Figure 6F p-AKT

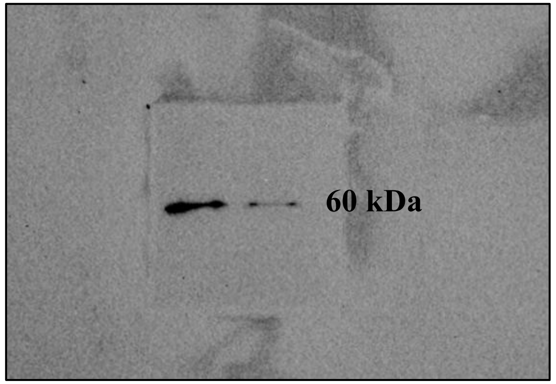

Figure 6F p-PI3K

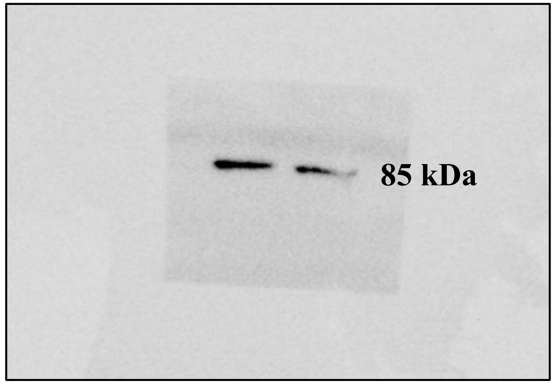

Figure 6F mTOR

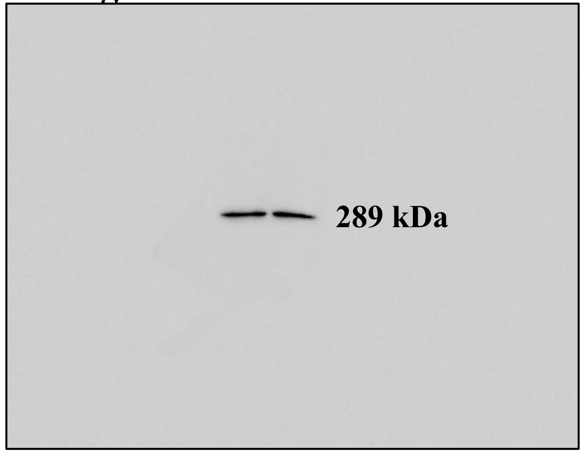

Figure 6F AKT

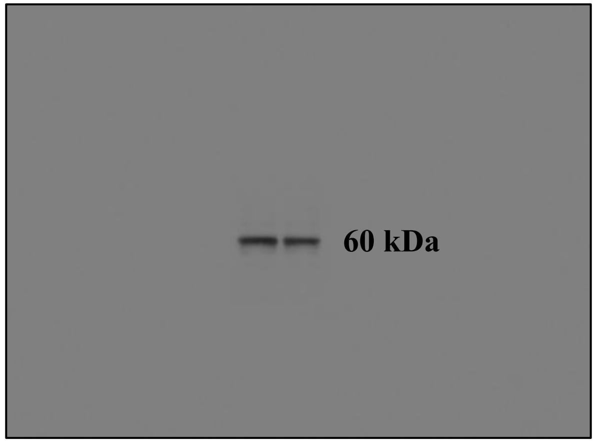

Figure 6F PI3K

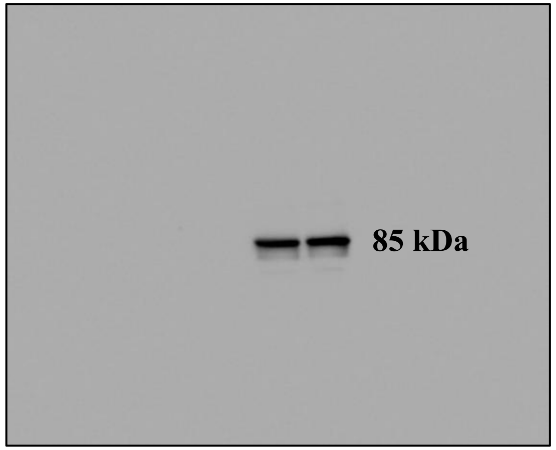

Figure 6F IGFBP1

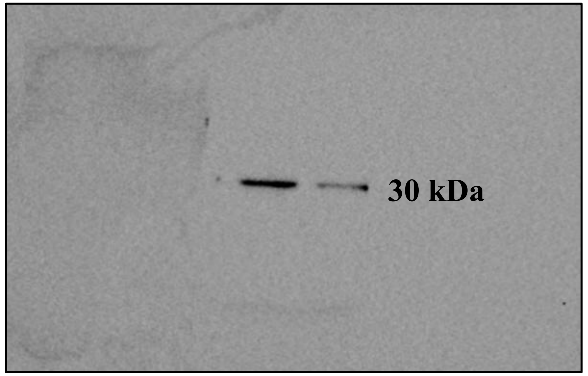

Figure 6F SIRT2

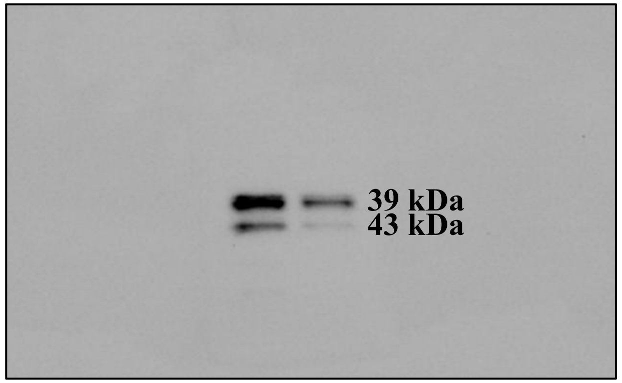

Figure 6F GAPDH

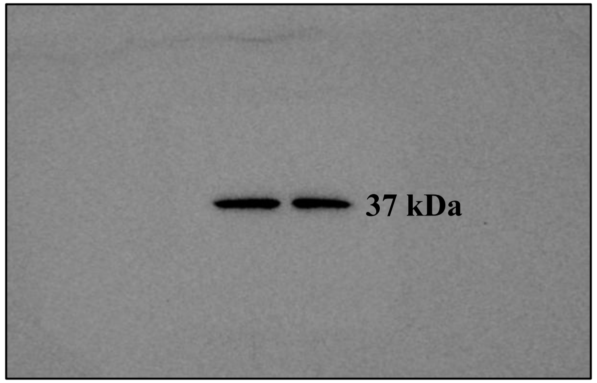

Original data of Figure 6

Figure 6H p-mTOR

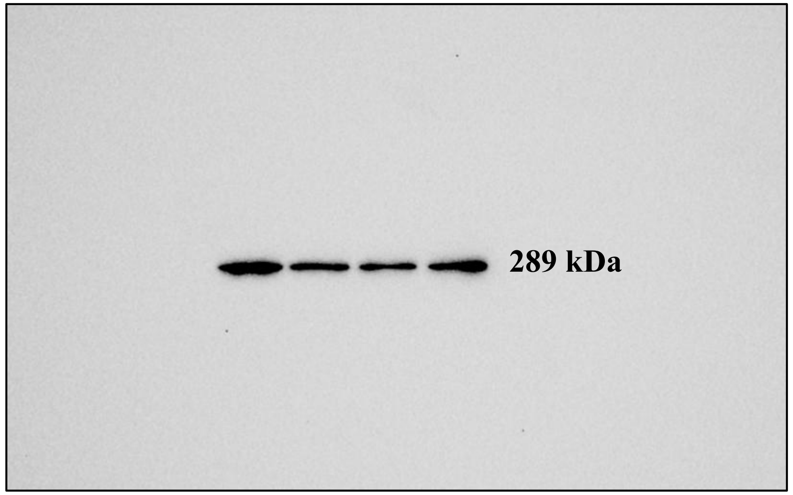

Figure 6H p-AKT

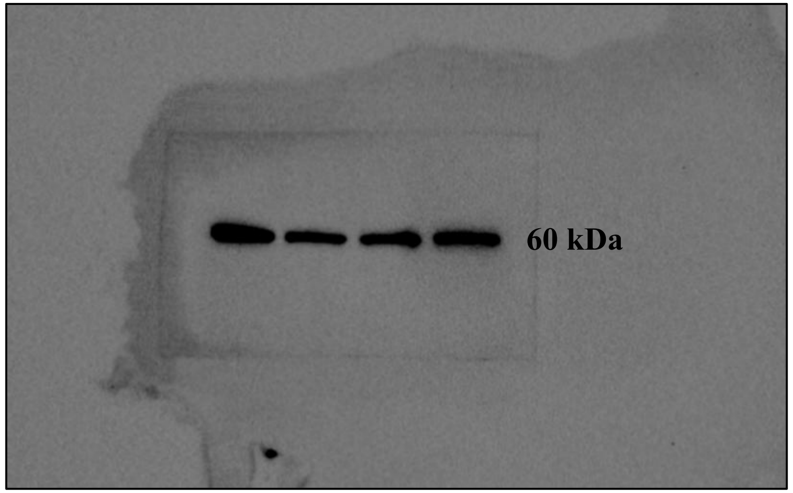

Figure 6H p-PI3K

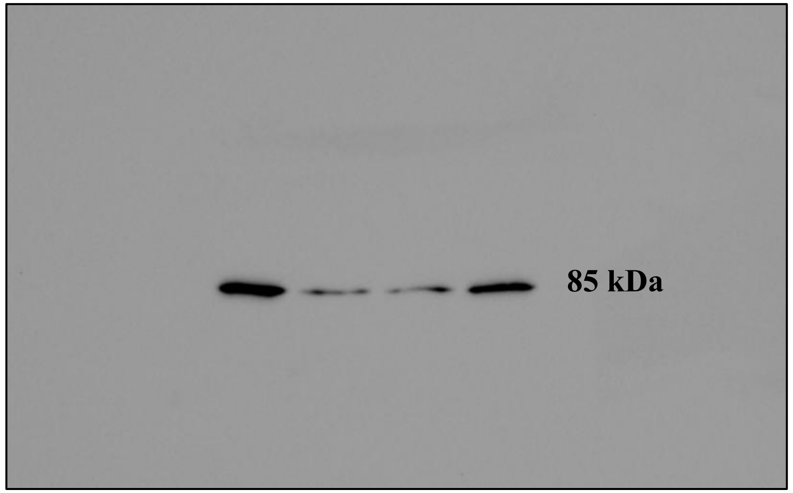

Figure 6H mTOR

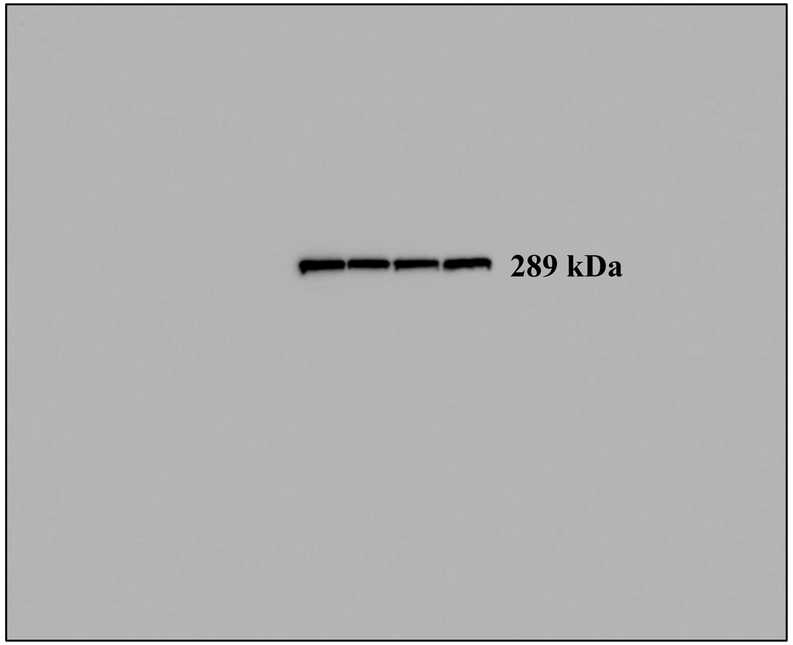

Figure 6H AKT

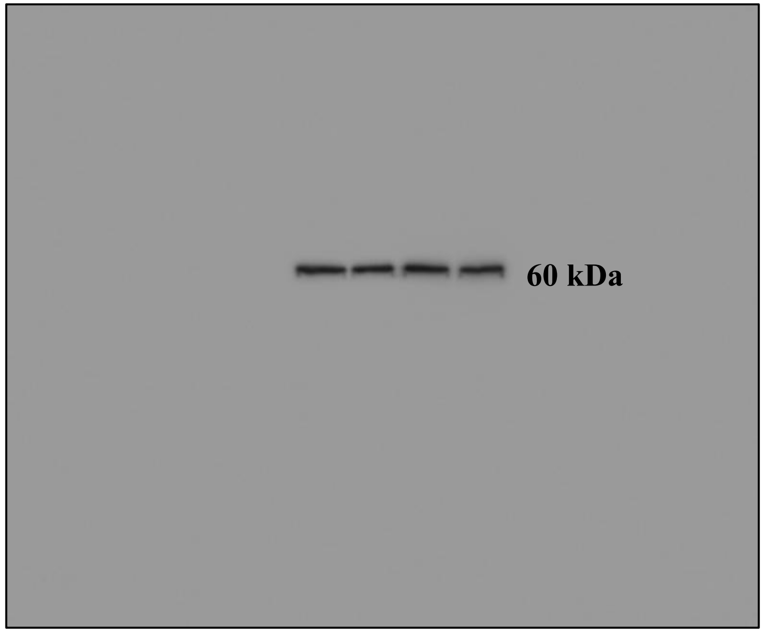

Figure 6H PI3K

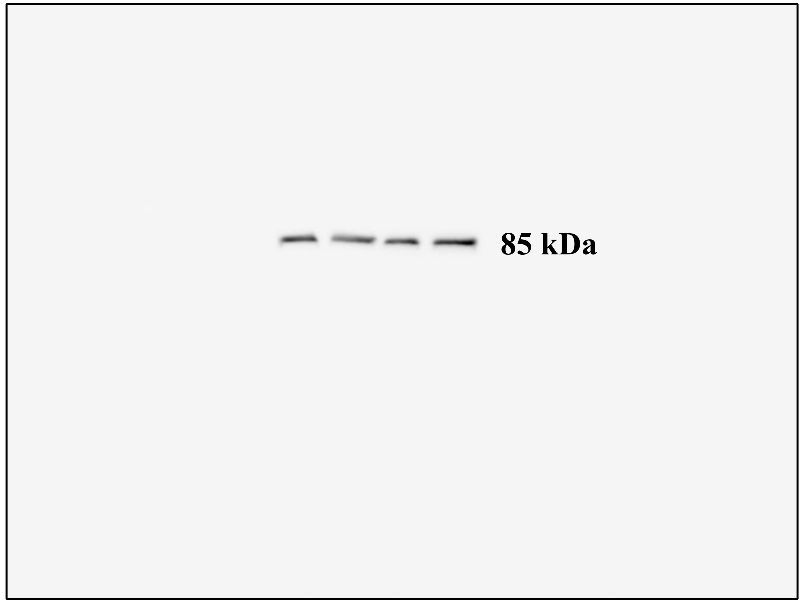

Original data of Figure 6

Figure 6H IGFBP1

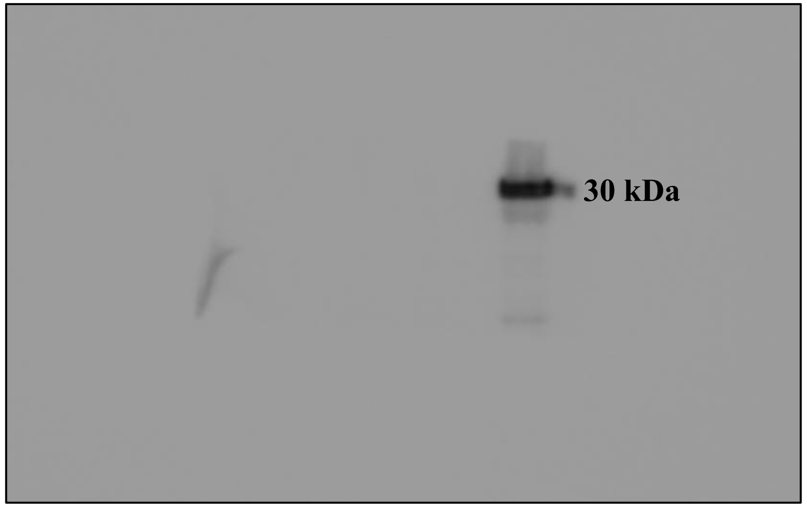

Figure 6H SIRT2

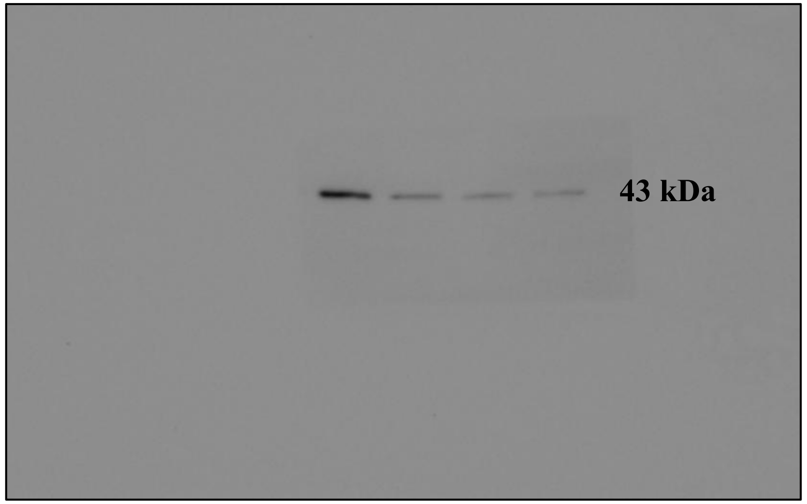

Figure 6H GAPDH

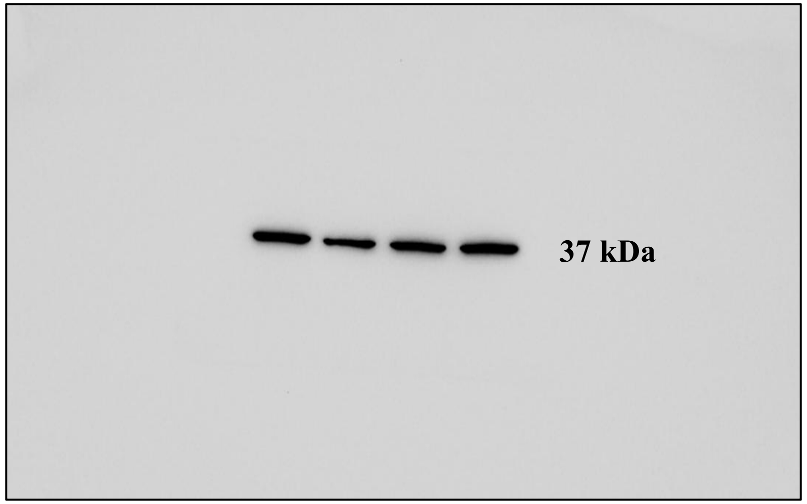

Original data of Figure 6

Figure 6I Ac-α-tubulin（Lys40）

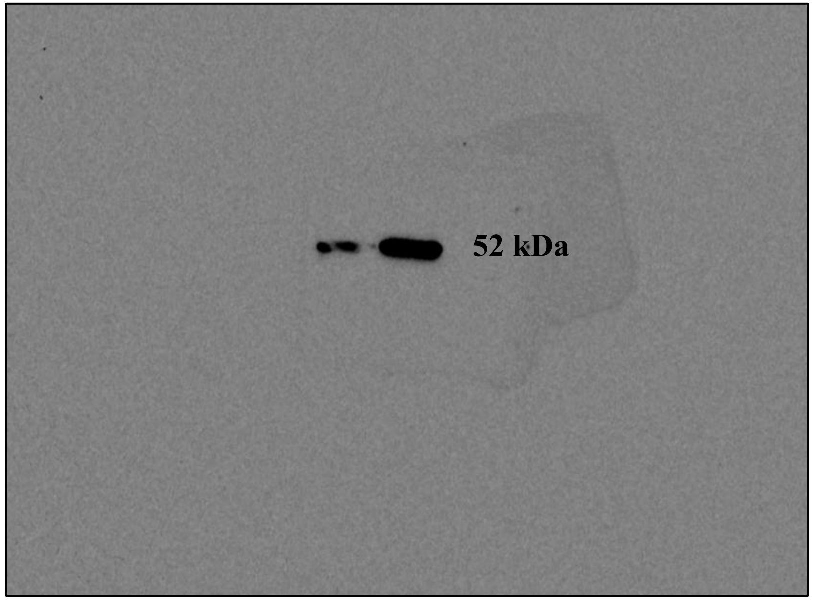

Figure 6I α-tubulin

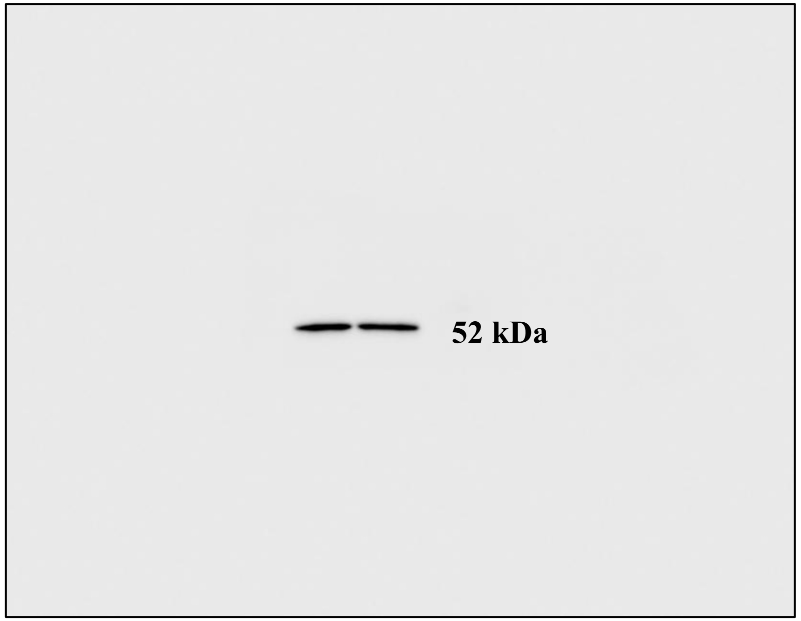

Figure 6I Acetyl-NF-κB p65 (Lys310)

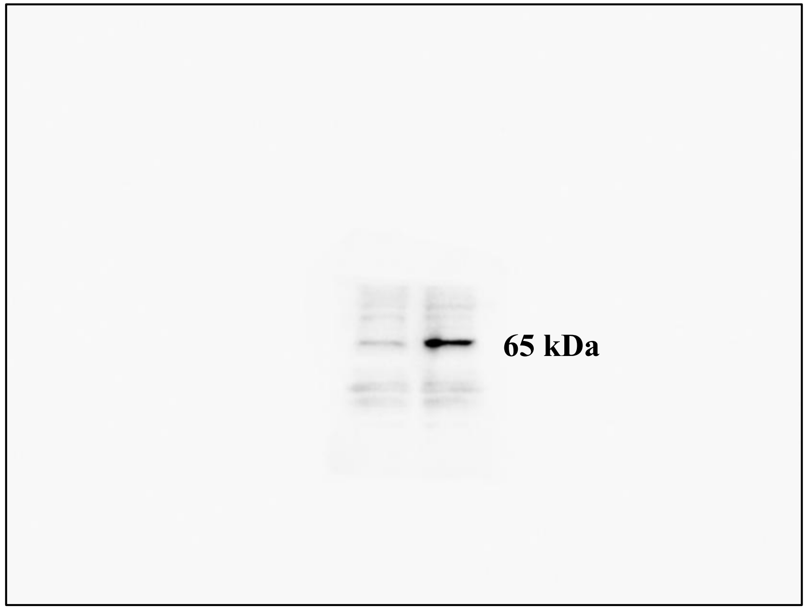

Figure 6I NF-κB p65

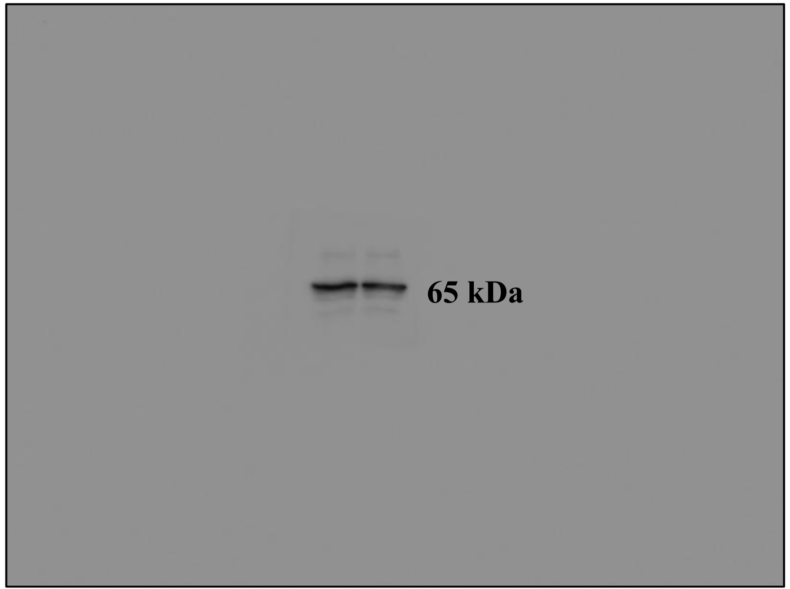

Figure 6I SIRT2

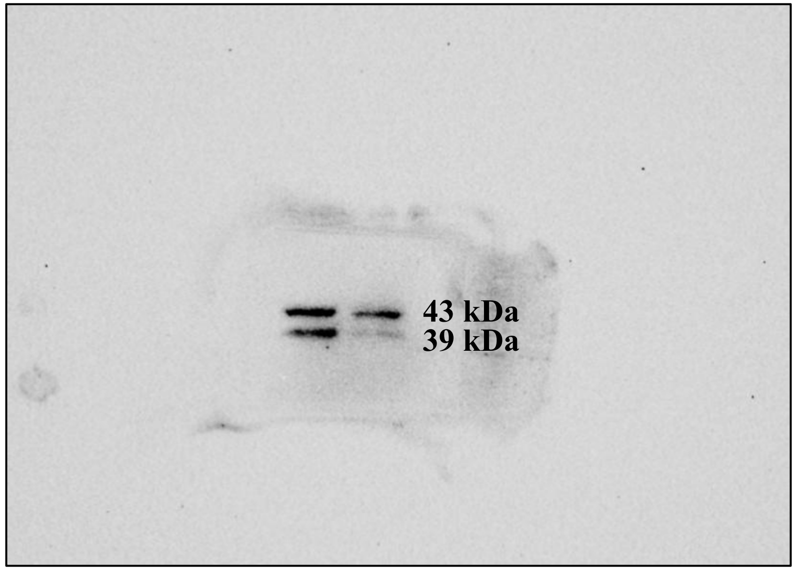

Figure 6I GAPDH

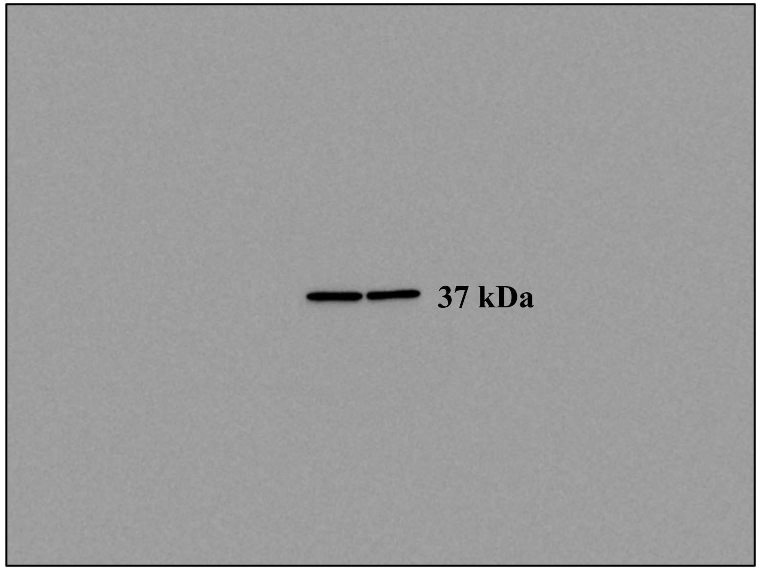

Supplement: Supplementary file 1 — Supplementary Material 1. [file 41598_2026_49240_MOESM1_ESM.pdf]
