# Supplementary material for: Dihydroartemisinin exerts antitumour activity by blocking SIRT2-IGFBP1-induced PI3K/AKT/mTOR signal transduction in liver cancer
Source: Sci Rep. 2026 Apr 20;16:18299. doi: 10.1038/s41598-026-49240-5 (PMC13260731; doi:10.1038/s41598-026-49240-5)
Supplement: Supplementary file 3 — Supplementary Material 3. [file 41598_2026_49240_MOESM3_ESM.docx]

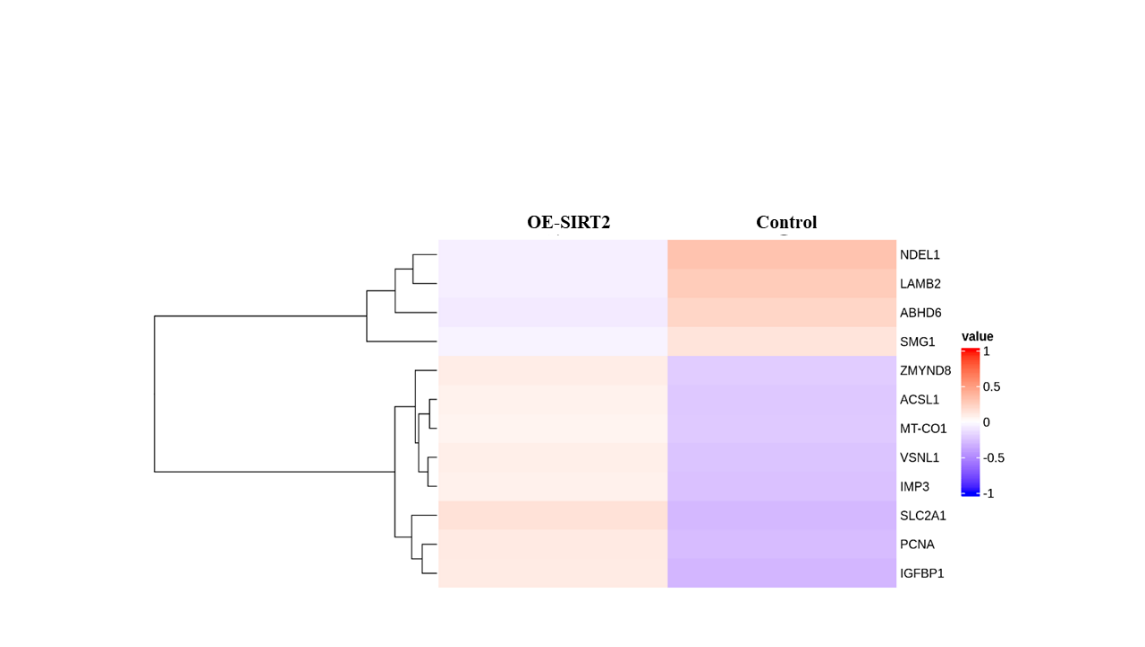


**Supplementary Figure S1:** Proteomic analysis was performed to study the molecular mechanism of SIRT2 in hepatocellular carcinogenesis. NDE-like 1 (NDEL1); suppressor of morphogenesis in genitalia 1 (SMG1); alpha/beta-hydrolase domain-containing 6 (ABHD6); Laminin subunit beta-2 (LAMB2); solute carrier family 2 member 1 (SLC2A1); IGFBP1, proliferating cell nuclear antigen (PCNA); Visinin-like 1 (VSNL1); insulin-like growth factor 2 mRNA binding protein 3 (IMP3); Zinc Finger MYND-Type Containing 8 (ZMYND8); Acyl CoA synthase long-chain family member 1 (ACSL1); mitochondrial cytochrome c oxidase subunit-1 (MT-CO1).


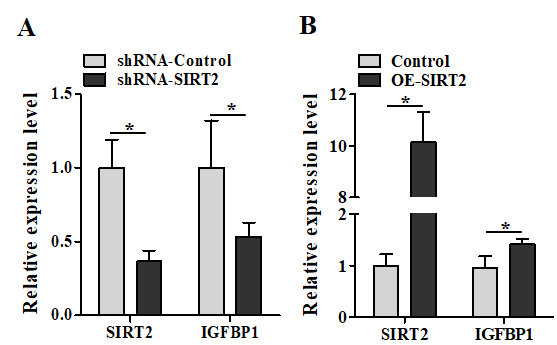


**Supplementary Figure S2:** SIRT2 regulates IGFBP1 expression at the transcriptional level. (A) The relative mRNA expression levels of SIRT2 and IGFBP1 in liver cancer cells transduced with SIRT2-specific shRNA (shRNA-SIRT2) or negative control shRNA (shRNA-Control) were measured by RT-qPCR. (B) The relative mRNA expression levels of SIRT2 and IGFBP1 in liver cancer cells transfected with the SIRT2 overexpression vector (OE-SIRT2) or empty vector (Control) were measured by RT-qPCR. GAPDH was used as the internal reference. Data are presented as the mean ± SD (n = 3 independent experiments). ***** *p* < 0.05 compared with the corresponding control group, Student’s t-test.


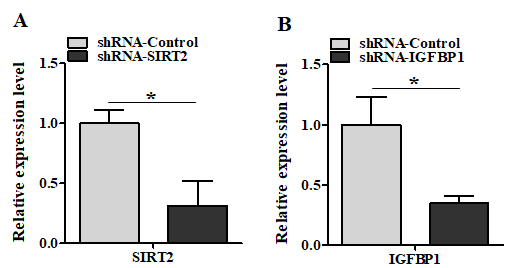


**Supplementary Figure S3:** Confirmation of SIRT2 and IGFBP1 knockdown efficiency at the mRNA level. (A) The relative mRNA expression of SIRT2 in HepG2 cells transduced with SIRT2-specific shRNA (shRNA-SIRT2) or negative control shRNA (shRNA-Control) was measured by RT-qPCR. (B) The relative mRNA expression of IGFBP1 in HepG2 cells transduced with IGFBP1-specific shRNA (shRNA-IGFBP1) or negative control shRNA (shRNA-Control) was measured by RT-qPCR. GAPDH was used as the internal reference. Data are presented as the mean ± SD (n = 3 independent experiments). ****** *p* < 0.01 compared with the control group, Student’s t-test.


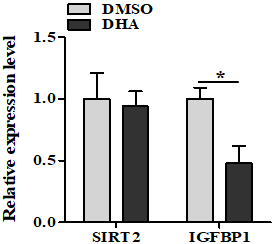


**Supplementary Figure S4:** Effect of DHA on the mRNA expression of SIRT2 and IGFBP1. HepG2 cells were treated with DMSO (control) or DHA. The relative mRNA expression levels of SIRT2 and IGFBP1 were measured by RT-qPCR. Data are presented as the mean ± SD (n = 3 independent experiments). ****** *p* < 0.01 compared with the control group, Student’s t-test.

**Supplementary Table S1:** Complete list of identified and differentially expressed proteins from the TMT-based quantitative proteomic analysis.
